# Toxoplasmocidal and Cytotoxic Activities Guided Isolation and Characterization of an Undescribed Bioflavonoid-di-*C*-glucoside from *Cycas rumphii* Miq. Cultivated in Egypt

**DOI:** 10.3390/plants11212867

**Published:** 2022-10-27

**Authors:** Hosam M. El-Seadawy, Kamilia A. Abo El-Seoud, Mona El-Aasr, Haytham O. Tawfik, Amany E. Ragab

**Affiliations:** 1 Department of Pharmacognosy, Faculty of Pharmacy, Tanta University, Tanta 31527, Egypt; 2 Department of Pharmaceutical Chemistry, Faculty of Pharmacy, Tanta University, Tanta 31527, Egypt

**Keywords:** *Cycas rumphii*, Cycadaceae, toxoplasmosis, cytotoxicity, phenolic acid, biflavonoids, 4′, 4′′′ biapigenin di-C-glucoside

## Abstract

Toxoplasmosis and cancer are serious worldwide diseases, and the available drugs cause serious side effects. Investigation for new alternative therapies from natural sources is now an increasing concern. Herein, we carried out, for the first time, an in vitro screening of *Cycas rumphii* Miq. leaves for toxoplasmocidal effect, using *Viruluent RH Toxoplasma gondii,* and cytotoxic activity against HEPG-2, HCT-116 and HELA cancer cell lines using MTT assay. Among the tested extracts, the ethyl acetate fraction was the most effective against *T. gondii*, with an EC_50_ of 3.51 ± 0.2 µg/mL compared to cotrimoxazole (4.18 ± 0.01 µg/mL) and was the most potent against the tested cell lines, especially HEPG-2, with an IC_50_ of 6.98 ± 0.5 µg/mL compared to doxorubicin (4.50 ± 0.2 µg/mL). Seven compounds were isolated from the ethyl acetate fraction by extensive chromatographic techniques and fully elucidated using different spectroscopies. Compound (**7**) is an undescribed 4′, 4′′′ biapigenin di-C-glucoside, which showed a strong cytotoxic activity. Four known biflavonoids (**1**, **2**, **4** and **5**) in addition to a phenolic acid ester (**3**) and a flavonoid glycoside (**6**) were also isolated. Compounds (**1**, **3** and **6**) were reported for the first time from *C. rumphii*.

## 1. Introduction

Toxoplasmosis is caused by *Toxoplasma gondii*, which is an obligate parasite [1]. The life cycle of this parasite occurs only in definitive hosts, such as domestic cats and wild felids, which pass the infection to humans [2]. The seroprevalence of this parasite in humans ranges from 30% in America and Europe to 60% in Africa [3]. This infection is a serious danger to immunocompromised people, leading to death, and to pregnant women, causing congenital toxoplasmosis and abortion [4].

The first treatment line of toxoplasmosis includes the combination of pyrimethamine, sulfadiazine and folic acid. However, pyrimethamine causes teratogenicity and other serious side effects [5,6].

Cancer is one of the prevalent diseases worldwide causing death, therefore attracting the interest of scientists to deliver new pipelines for treatment and to lower or avoid the known side effects of the currently used drugs.

The genus *Cycas* is a rich source of flavonoids, biflavonoids, phenolic acids, tannins, lignans, fatty acids and sterols, which exhibit a plethora of biological activities [7,8,9]. *Cycas rumphii* Miq. is a member of the cycadaceae family native to Indonesia [10]. It is traditionally used in Southeast Asian countries, such as India, Bangladesh and Indonesia for the treatment of wounds, ulcers, boils, itchy skin lesions and sore throat. In addition, it is used for nephrotic pain, headache, bloody vomiting and flatulence [11,12]. The plant extract showed a potential antimicrobial activity against a range of human pathogenic bacteria [8]. There are no reports on the phytochemicals of *Cycas rumphii* Miq. having antitoxoplasma and cytotoxic properties. Thus, we aimed to evaluate the toxoplasmocidal and cytotoxic potentials of *Cycas rumphii* Miq. and to isolate and characterize the phytochemicals which could be responsible for the resulting activity.

## 2. Results and Discussion

### 2.1. Biological Activity

#### 2.1.1. Toxoplasmocidal Activity

**Figure 1:**
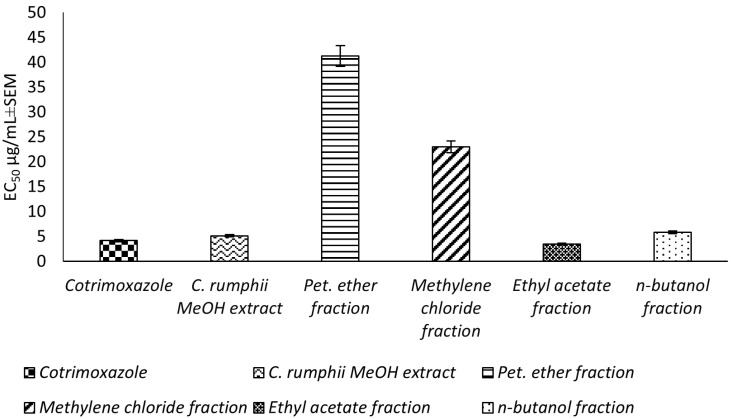
Toxoplasmocidal effect (EC_50_ ± SEM) of *C. rumphii* methanol extract and its different fractions against *T. gondii*.

This study reports, for the first time, an in vitro evaluation for the potential toxoplasmocidal activity of *C. rumphii* leaves against *T. gondii* RH strain tachyzoites. The total methanol extract of the *C. rumphii* leaves exhibited an EC_50_ of 5.15 ± 0.3 μg/mL, while the positive control drug (cotrimoxazole) showed an EC_50_ value of 4.18 ± 0.3 μg/mL. These results motivated us to investigate the toxplasmocidal activity of the different prepared fractions from the methanol extract. Amongst the tested extracts, the ethyl acetate fraction was the most potent, with an EC_50_ of 3.51 ± 0.2 μg/mL, which is lower than that of cotrimoxazol (Figure 1, Supplementary material Table S1). The results indicated a promising toxoplasmocidal activity of *C. rumphii* leaves against *T. gondii* RH strain.

#### 2.1.2. Cytotoxic Activity

**Figure 2:**
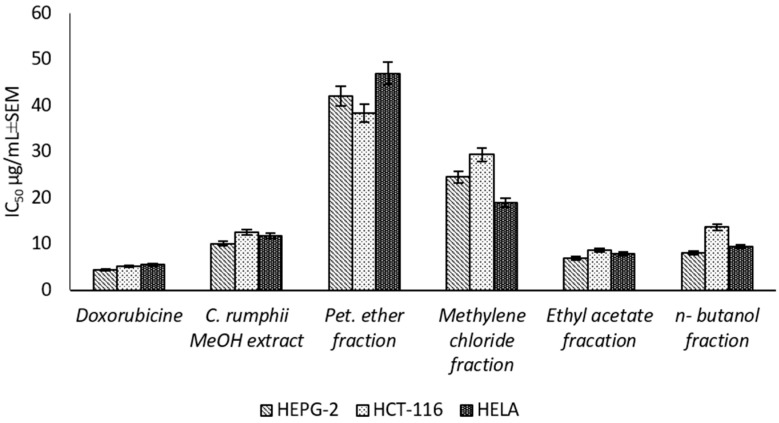
Cytotoxic effect (IC_50_ ± SEM) of *C. rumphii* methanol extract and different fractions against different cell lines.

The present study is the first report for assessing the cytotoxic activity of the *C. rumphii* leaves against six different cell lines (Supplementary Material Table S2). The results showed that the total methanol extract had a strong cytotoxic activity against HEPG-2, HELA and HCT-116 with IC_50_ values of 10.09 ± 0.9, 11.79 ± 1.0 and 12.58 ± 1.1 μg/mL, respectively, according to the classification of Hossan and Abu Melha, 2014 [13] (Figure 2). Interestingly, the total methanol extract showed an IC_50_ value of 53.72 ± 3.7 μg/mL against normal cell line (WISH), while the positive control (doxorubicin) exhibited an IC_50_ value of 7.79 ± 0.5 μg/mL. Thus, the methanol extract of *C. rumphii* leaves was considered safer on normal cells and more specific to cancer cells than doxorubicin. The different fractions of the methanol extract were tested against the most affected cell lines (HEPG-2, HELA and HCT-116). The results revealed that the ethyl acetate fraction was the most potent amongst the tested fractions with very strong cytotoxic activity. This fraction exhibited IC_50_ values of 6.98 ± 0.5, 7.94 ± 0.8 and 8.70 ± 0.9 μg/mL against the HEPG-2, HELA and HCT-116 cell lines, respectively (Figure 2, Supplementary material Table S3). According to the biological evaluation, the ethyl acetate fraction showed the highest activity. Thus, phytochemical investigation of this fraction was carried out to isolate and characterize the compounds mediating the exhibited biological potential.

### 2.2. Phytochemical Investigation

**Figure 3:**
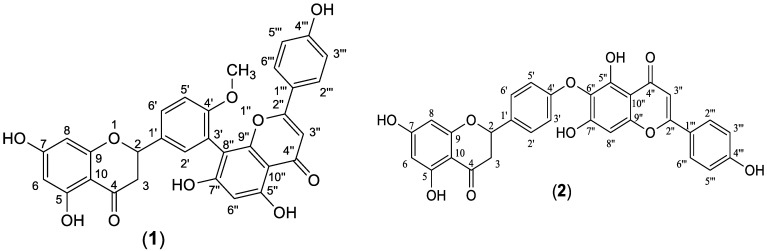

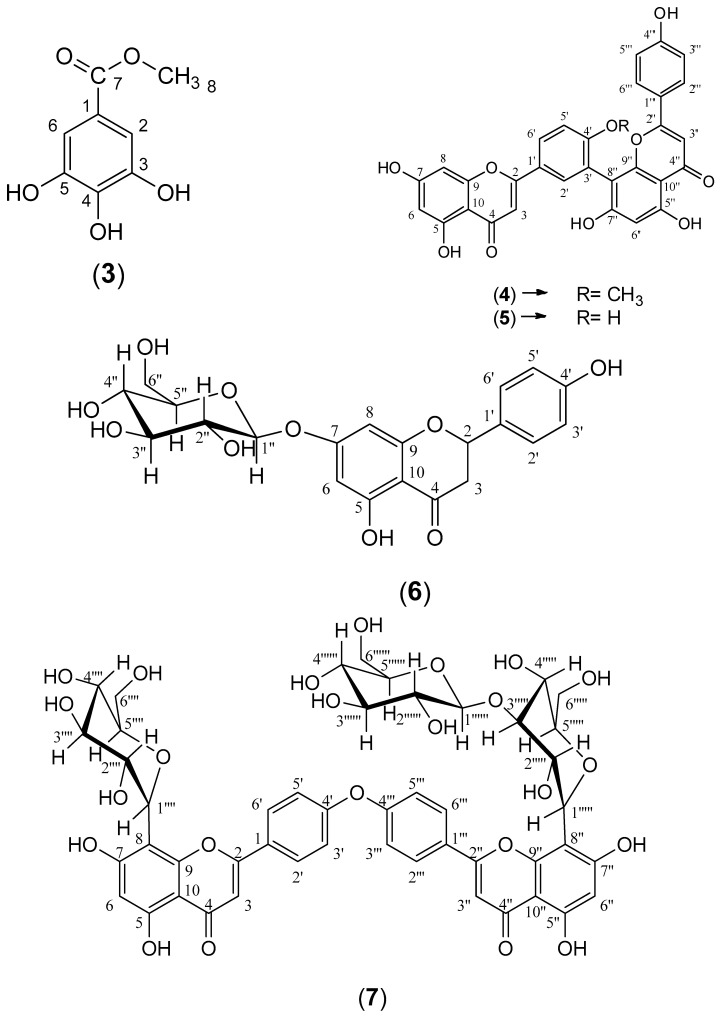
Structures of compounds (**1**–**7**) isolated from the ethyl acetate fraction of *C. rumphii* Miq.

The ethyl acetate fraction was subjected to extensive chromatographic separations to isolate seven compounds (**1**–**7**) (Figure 3). Six known compounds were 2,3-dihydro-4′-*O*-methylamentoflavone (**1**) [14,15], 2,3-dihydrohinokiflavone (**2**) [15,16,17], methyl gallate (**3**) [18,19], amentoflavone 4′-*O*-methyl ether (**4**) [15,20,21], amentoflavone (**5**) [15,22] and naringenin 7-*O*- *β*-D glucoside (**6**) [18,23,24]. Compound (**7**) was identified as a novel 4′, 4′′′ biapigenin di-C-glucoside. Compounds (**1**, **3** and **6**) were isolated for the first time from *C. rumphii*. The structures of the compounds (**1**–**6**) were identified by various spectroscopic analyses, including (UV, IR, ESIMS, ^1^H, ^13^C, DEPTQ, HSQC and HMBC NMR), in addition to the comparison with the available authentic compounds and the literature data [14,15,16,17,18,19,20,21,22,23,24]. The elucidation of compound (**7**) is discussed in detail below. The IR, UV, mass and NMR spectra of all the isolated compounds are presented in (Supplementary Material Figures S1–S29).

#### Identification of Compound (**7**)

**Figure 4:**
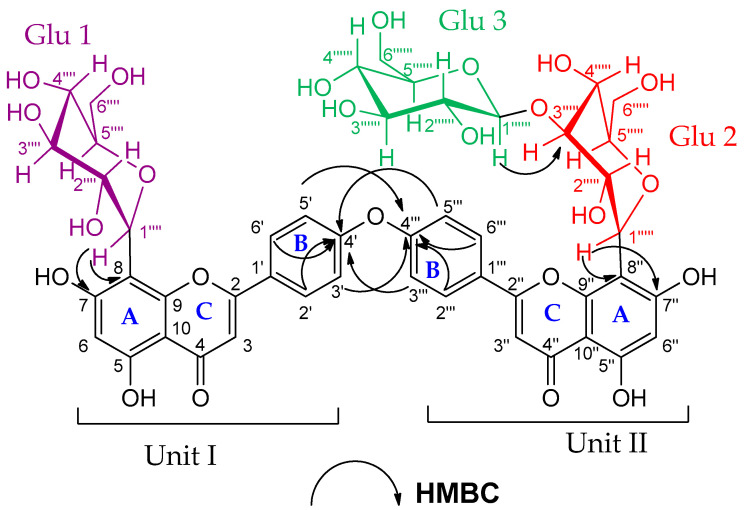
HMBC correlations of compound (**7**). (4′, 4′′′ biapigenin-8″-C-*β*-D-glucopyranosyl [1′′′′′′→3′′′′′] *β*-D-glucopyranoside-8-C-*β*-D-glucopyranoside).

Compound (**7**) was obtained as an amorphous dark brown powder. It gave a yellow color with 5% AlCl_3_, a brown color with 10% H_2_SO_4_ and UV λ_max_ at 238, 268 and 324 nm, suggesting that compound (**7**) is a flavonoid glycoside. The ^1^H-NMR data of compound (**7**) (Supplementary Material Figure S26A) showed two sets of AA′BB′ coupling systems at δ_H_ 6.95 (4H, d, *J* = 8 Hz), 7.85 (2H, d, *J* = 8 Hz) and 7.99 (2H, d, *J* = 8 Hz), which indicated the presence of two 1, 4 di-substituted aromatic rings, suggesting a biflavonoid structure consisting of two units (I and II, see Figure 4). In addition, the ^1^H-NMR spectrum showed two broad singlets at δ_H_ 6.62 and δ_H_ 6.27, each integrating for two protons. These signals indicated that ring A, in both units (I and II), is trisubstituted. The DEPTQ-NMR spectrum (Supplementary Material Figure S26B) showed two carbonyl signals at δ_dept-Q_ 182.7 and 182.8 assigned to C-4 and C-4″, respectively, which confirmed the biflavonoid structure of compound (**7**). In addition to the carbonyl signals, nine oxygen-bearing quaternary carbon signals were indicated. Based on the HSQC and HMBC correlations (Supplementary Material Figure S27), four carbons were connected to hydroxyl groups at δ_dept-Q_ 161.2, 163.26, 161.5 and 163.5 (C-5, C-7, C-5″ and C-7″), respectively, and another four carbons were connected to pyranone oxygen at δ_dept-Q_ 164.8, 156.2, 165.1 and 156.8 (C-2, C-9, C-2″ and C-9″), respectively [25,26], thus proposing two apigenin units. The remaining oxygen-bearing quaternary carbon signal at δ_dept-Q_ 161.3 (C-4′, C-4′′′) suggested that C-4′ and C-4′′′ were involved in the interflavonoid linkage of the two apigenin units. The HMBC spectral data are consistent with C4′-O-C4′′′ linkage through the correlation of H-3′, 5′ and C-4′′′, and the correlation between H-3′′′, 5′′′ and C-4′ (Supplementary Material Figure S27B). The ^1^H-NMR and DEPTQ-NMR data of the aglycon part were comparable to the literature data of loniflavone with the exception of small differences in the chemical shifts of (C-2′, C-6′, C-3′, C-5′ and C-4′) due to the hydroxyl group at C-3′ position in loniflavone moiety [25,26]. The ^1^H-NMR spectrum showed three anomeric doublets at δ_H_ 5.14 (1H, d, *J* = 9.2 Hz, H-1′′′′), 5.07 (1H, d, *J* = 9.6 Hz, H-1′′′′′) and 4.21 (1H, d, *J* = 7.6 Hz, H-1′′′′′′), which suggested the presence of three sugar moieties (Glu-1, Glu-2 and Glu-3). This was confirmed by DEPTQ-NMR spectrum, which showed 18 carbon signals related to three glucose moieties [27]. The configuration of the three glucose moieties is *β* as revealed by the high coupling constant values of 9.2, 9.6 and 7.6 Hz for Glu-1, Glu-2 and Glu-3 anomeric protons, respectively. The acid hydrolysis of compound 7 resulted in the hydrolysis of only the *O*-glucoside bond, and the resulting sugar was identified as *β*-D-glucose through paper chromatography alongside an authentic *β*-D-glucose. For C-glucoside, there is no reliable method for hydrolysis of such bond; thus, the configuration of glucose at these bonds was determined as *β* based on the large *J* value for the anomeric protons (9.2 and 9.6 Hz) [28,29] and by investigating the carbon resonances for Glu-1 and Glu-2, HMBC and HSQC correlations compared to the published data of vicenin-2 which also has glucose molecules at C-6 and C-8 positions linked through C-bond as in compound (**7**) [29]. The position of the glucose moieties was suggested by the absence of *meta*-coupled proton signals of ring A in units I and II. There is only one singlet at δ_H_ 6.27 assigned for H-6, H-6″ in addition to the downfield shift of Δ ≅ 9 ppm of C-8 and C-8″ (δ_dept-Q_ 103.0 and 103.6, respectively) compared to the apigenin ^13^C-NMR spectral data [15], which suggested that C-8 and C-8″ were linked to sugar moieties with C-C glycosidic linkage. This was confirmed by the HMBC analysis (Figure 4), which indicated the correlations between the Glu-1 anomeric proton at δ_H_ 5.14 and the carbons C-8 and C-7 at δ_dept-Q_ 103.0, 163.2, respectively. In addition, the Glu-2 anomeric proton at δ_H_ 5.07 showed correlations to C-8″ and C-7″ at δ_dept-Q_ 103.6 and 63.5, respectively. The remaining glucose moiety (Glu-3) was linked with ether linkage to Glu-2 (at C-3′′′′′) as confirmed by the HMBC correlation between the Glu-3 anomeric proton at δ_H_ 4.21 and Glu-2 (at C-3′′′′′) at δ_dept-Q_ 80.2. Also, the downfield shift of Δ ≅ 4 ppm at C-3′′′′′ (δ_dept-Q_ 80.2) indicated that C-3′′′′′ is linked to C-1′′′′′′. The HRMS (ESI) spectrum of compound (**7**) (Supplementary Material Figure S28) indicated a pseudomolecular ion at m/z 1009.26259 [M+H]^+^ for a molecular formula of C_48_H_49_O_24_ (calculated: 1009.26138), which supported the results. The spectral data confirmed that compound (**7**) is 4′,4′′′ biapigenin-8″-C-*β*-D-glucopyranosyl [1′′′′′′→3′′′′′] *β*-D-glucopyranoside-8-C-*β*-D-glucopyranoside (Figure 4). The specific rotation ${\left[\alpha \right]}_{D}^{20}\;$of compound (**7**) is +36.45 (C 0.1, methanol). To our knowledge, this is the first report of 4′-*O*-4′′′ biapigenin di-C-glucosides from plants.

### 2.3. Biological Activities of Pure Compounds

#### Cytotoxic Activity

**Figure 5:**
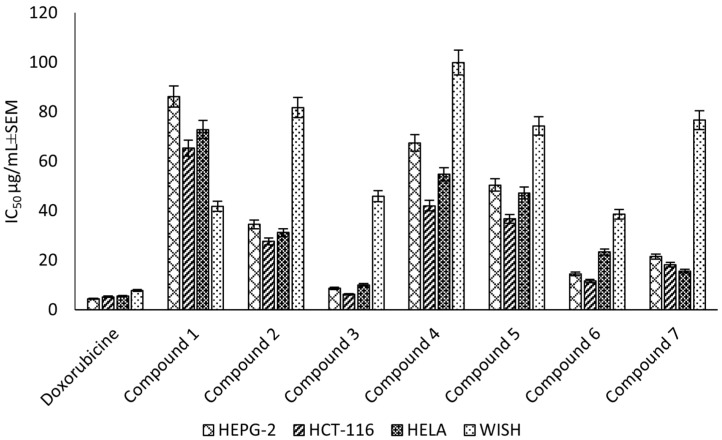
Cytotoxic effect (IC_50_ ± SEM) of *C. rumphii* isolated compounds against the HEPG-2, HCT-116 and HELA cell lines.

Compounds (**1**–**7**) were evaluated for the cytotoxic activity against the HEPG-2, HELA and HCT-116 cell lines. The results showed that compound (**3**) was the most potent against HEPG-2, HELA and HCT-116, with IC_50_ values of 8.67 ± 0.6, 10.08 ± 0.9 and 6.24 ± 0.5 μg/mL, respectively, followed by compound (**6**), which exhibited a strong cytotoxic activity, with IC_50_ values of 14.49 ± 1.1, 23.32 ± 2.0 and 11.63 ± 0.9 μg/mL, respectively. Finally, compound (**7**) showed a strong cytotoxic activity, with IC_50_ values of 21.47 ± 1.8, 15.66 ± 1.3 and 18.17 ± 1.4 μg/mL, respectively (Figure 5). Additionally, the selectivity indices for the ethyl acetate fraction and its constituents against these cancer cell lines (HEPG-2, HELA and HCT-116) were calculated as reported in the literature data [30] (Supplementary Material Table S4). The higher the magnitude of the selectivity index of a test material, the greater its selectivity. In this study, the ethyl acetate fraction and its isolated compound (**3**) showed higher selectivity for the cancer cells than the normal cells compared to doxorubicin.

## 3. Materials and Methods

### 3.1. General Experimental Procedures

Solvents used were of HPLC analytical grade ≥99.9% and were purchased from Sigma Co. (St. Louis, MI, USA)**.** RPMI-1640 medium reagent, 4,5 dimethylthiazole-2-yl-2,5 diphenyltetrazolium bromide (MTT)***,*** dimethyl sulfoxide (DMSO), doxorubicin HCl, phosphate buffer saline and trypan blue were obtained from Sigma Co. (St. Louis, MI, USA). Fetal bovine serum was purchased from Gibco Co. (Carlsbad, CA, USA) and cotrimoxazole (Septrin™ oral suspension) from GlaxoSmithKline.

NMR experiments were performed using a Bruker Avance III spectrometer (Rheinstetten, Germany), with 400 MHz for ^1^H and 100 MHz for ^13^C and DEPT-Q NMR. ESI-MS spectra were recorded by Advion compact mass spectrometer (CMS) (New York, NY, USA). High-resolution mass spectra were measured using the Q-TOF-LC/HRMS system (6530) from Agilent Technologies Co. (Waldbronn, Germany) equipped with an autosampler (G7129A) with an ESI ionization source. Melting point determination was carried out using a Gallenkamp melting point apparatus from Hanon Co. (Jinan, China). Optical rotation was measured using a Polax-2L Polarimeter (Atago Co., Tokyo, Japan). UV spectra were recorded using a UV/Vis spectrophotometer (UV-1800 from Shimadzu Co., Tokyo, Japan). IR spectra were measured as KBr discs using an FT/IR-6100 spectophotometer from Jasco Co. (Tokyo, Japan). An ELISA Processor II Microplate Reader EXL800 from Biotek Co. (Winooski, VT, USA) was used for the cytotoxic assessment.

Silica gel (70–230 mesh) and precoated TLC sheets of silica gel F_254_ were purchased from Merck Co. (Darmstadt, Germany), while Sephadex LH-20 was obtained from Sigma-Aldrich Chemical Co. (St. Louis, MI, USA). Authentic samples of amentoflavone and bilobetin for Co-TLC and IR fingerprint experiments were provided by the Department of Pharmacognosy, Faculty of Pharmacy, Tanta University, Egypt. AlCl_3_ (5%) and 10% H_2_SO_4_ spray reagents were used for detection on TLC. Solvent systems used for TLC were CH_2_Cl_2_-MeOH (9:1) “S1”, CH_2_Cl_2_-MeOH (8:2) “S1” and CH_2_Cl_2_-MeOH-H_2_O (7:3:0.5) “S3”.

### 3.2. Plant Material

The leaves of *C. rumphii* Miq. were collected from El-Abd Garden at 68 kilos from the desert Cairo-Alexandria Road in July 2018. It was kindly provided and identified by the researcher Rabea Sharawy (Agronomist and palm researcher). A voucher sample (No. PGG-012) was deposited at the herbarium of the Department of Pharmacognosy, Faculty of Pharmacy, Tanta University, Egypt.

### 3.3. Extraction and Isolation

The plant material was dried in shade, reduced to powder and stored in tightly closed containers. The plant powder (5 Kg) was extracted with methanol by cold maceration till exhaustion. The total methanol extract was evaporated under reduced pressure at 40 °C to yield a green residue (294 g). The methanol extract residue (274 g) was suspended in 50% aqueous methanol (1.5 L) and was successively fractionated with petroleum ether (40–60 °C), methylene chloride, ethyl acetate and *n*-butanol to yield 38.03 g, 8.10 g, 20.1 g and 59.20 g, respectively.

The ethyl acetate fraction (10 g) was chromatographed on a silica gel column (Φ 4.5 cm × 32 cm, 300 g) using a gradient elution, starting with pure CH_2_Cl_2_, and the polarity was increased using MeOH. Fractions (50 mL) were collected, and similar fractions, on TLC, were combined to afford five groups of fractions (F1 to F5). F1, eluted with CH_2_Cl_2_:MeOH (95:5), gave a yellow-colored residue (135 mg), which was chromatographed further on a silica gel column (Φ 1.5 cm × 10 cm, 10 g) using a gradient elution of CH_2_Cl_2_ and MeOH to obtain three subfractions F1-1 to F1-3. F1-1 (78 mg) was re-chromatographed on a Sephadex LH-20 column (Φ 1.5 cm × 25 cm, 20 g) using MeOH (HPLC grade) to give compound (**1**) (8 mg).

F2, eluted with CH_2_Cl_2_:MeOH (90:10), gave a brown-colored residue (342 mg), which was re-chromatographed on a silica gel column (Φ 1.5 cm × 22 cm, 14 g) using CH_2_Cl_2_ and MeOH in a gradient elution to give two subfractions F2-1 and F2-2. F2-1 (92 mg) was purified on a Sephadex LH-20 (Φ 1.5 cm × 25 cm, 20 g) using MeOH (HPLC grade) to give compound (**2**) (22 mg). F2-2 (125 mg) was also purified further on a Sephadex LH-20 (Φ 1.5 cm × 25 cm, 20 g) using MeOH (HPLC grade) to afford compounds (**3)** and (**4)** (11 and 16 mg, respectively).

F3, eluted with CH_2_Cl_2_:MeOH (85:15), yielded a yellow-colored residue (571 mg), which was chromatographed further on a silica gel column (Φ 1.5 cm × 23 cm, 15 g) using a gradient elution of CH_2_Cl_2_ and MeOH to afford three subfractions, F3-1 to F3-3. F3-3 (50 mg) was re-chromatographed on a Sephadex LH-20 column (Φ 1.5 cm × 25 cm, 20 g) using MeOH (HPLC grade) to afford compound (**5**) (38 mg).

F4, eluted with CH_2_Cl_2_:MeOH (80:20), produced a dark brown-colored sticky residue (1.3 g). Further chromatography on a silica gel column (Φ 1.5 cm × 59 cm, 41 g) using a gradient elution with CH_2_Cl_2_ and MeOH resulted in three subfractions, F4-1 to F4-3. F4-3 (140 mg) was purified on a Sephadex LH-20 column (Φ 1.5 cm × 25 cm, 20 g) using MeOH (HPLC grade) to give compound (**6**) (21 mg).

F5, eluted with CH_2_Cl_2_:MeOH (75:25), yielded a brown-colored residue (1.7 g). Further chromatography on a silica gel column (Φ 2.5 cm × 30 cm, 50 g) using the same gradient elution system afforded three subfractions, F5-1 to F5-3. F5-3 (130 mg) was purified on a Sephadex LH-20 column (Φ 1.5 cm × 25 cm, 20 g) using MeOH (HPLC grade) to afford compound (**7**) (42 mg).

Acid hydrolysis of compound (**7**):

The procedure in reference [28] was followed with some modifications. Compound **7** (1 mg) was dissolved in 2 N HCl/methanol mixture (1:1, 2 mL) and heated at 100 °C for 1 h. Then, the solution was evaporated to remove the residual methanol, and the left solution was neutralized with NaHCO_3_. The sugar in the hydrolysis product was detected by paper chromatography alongside authentic sample of *β*-D-glucose using the solvent system *n*-butanol:acetic acid:water (4:1:5).

*2,3-Dihydro-4′-O-methylamentoflavone* (**1**)**.** Amorphous yellow powder; UV (MeOH) λ_max_: 230, 294 and 331 nm see (Supplementary Material Figure S4); ^1^H-NMR (CD_3_OD, 400 MHz) δ (ppm) 7.50 (m, H-2′, 1H), 7.48 (m, H-6′, 1H), 7.38 (m, H-2′′′, 6′′′, 2H), 7.10 (dd, *J* = 2.8, 8.8 Hz, H-5′, 1H), 6.68 (m, H-3′′′, 5′′′, 2H), 6.51 (s, H-3″, 1H), 6.24 (s, H-6″, 1H), 5.80 (d, *J* = 2.4 Hz, H-8, 1H), 5.77 (d, *J* = 2.4 Hz, H-6, 1H), 5.37 (dd, *J* = 2.8, 13.2 Hz, H-2, 1H), 3.66 (s, OMe-4′, 3H), 3.07 (m, H-3*_ax_*, 1H), 2.68 (m, H-3*_equ_*, 1H) see (Supplementary Material Figure S1A); ^13^C-NMR (CD_3_OD, 100 MHz) δ (ppm) 196.2 (C-4), 182.8 (C-4″), 167.0 (C-7), 164.5 (C-5), 164.1 (C-2″), 163.4 (C-9), 161.9 (C-5″), 161.3 (C-7″), 160.8 (C-4′′′), 158.1 (C-4′), 154.8 (C-9″), 131.1 (C-2′), 130.7 (C-1′), 127.9 (C-2′′′, 6′′′), 127.8 (C-6′), 121.7 (C-1′′′), 121.1 (C-3′), 115.4 (C-3′′′, 5′′′), 110.7 (C-5′), 104.8 (C-8″), 103.8 (C-10″), 101.9 (C-3″), 101.7 (C-10), 98.3 (C-6″), 95.7 (C-6), 94.8 (C-8), 79.0 (C-2), 54.7 (4′-OCH_3_), 43.0 (C-3) see (Supplementary Material Figure S1B); HSQC and HMBC NMR (CD_3_OD) see (Supplementary Material Figure S2); ESIMS: m/z 577.1 for [M+Na]^+^ and 553.1 for [M-H]^−^ see (Supplementary Material Figure S3).

*2,3-Dihydrohinokiflavone* (**2**)**.** Amorphous yellowish white powder; UV (MeOH) λ_max_: 223, 274 and 333 nm see (Supplementary Material Figure S7); IR (KBr disc) ν_max_ = 3418, 2922, 2853, 1641, 1502, 1462, 1375, 1284, 1245, 1168, 1091, 1030, 912, 832, 733, 594, 569, 507, 248 cm^−1^ see (Supplementary Material Figure S8); ^1^H-NMR (CD_3_OD, 400 MHz) δ (ppm) 7.91 (d, *J* = 8.8 Hz, H-2′′′, 6′′′, 2H), 7.44 (d, *J* = 8.8 Hz, H-2′, 6′, 2H), 6.97 (d, *J* = 8.8 Hz, H-3′′′, 5′′′, 2H), 6.96 (d, *J* = 8.8 Hz, H-3′, 5′, 2H), 6.69 (s, H-3″, 1H), 6.66 (s, H-8″, 1H), 5.93 (d, *J* = 2 Hz, H-8, 1H), 5.90 (d, *J* = 2 Hz, H-6, 1H), 5.43 (dd, *J* = 2.4, 12.4 Hz, H-2, 1H), 3.14 (dd, *J* = 12.8, 17.2 Hz, H-3*_ax_*, 1H), 2.76 (dd, *J* = 2.8, 17.2 Hz, H-3*_equ_*, 1H) see (Supplementary Material Figure S5A); ^13^C-NMR (CD_3_OD, 100 MHz) δ (ppm) 196.1 (C-4), 182.7 (C-4″), 167.0 (C-7), 165.1 (C-2″), 163.3 (C-5), 162.5 (C-9), 161.4 (C-4′′′), 158.2 (C-4′), 158.0 (C-7″), 154.4 (C-9″), 154.3 (C-5″), 132.3 (C-1′), 128.1 (C-2′′′, 6′′′), 127.3 (C-2′, 6′), 125.9 (C-6″), 121.7 (C-1′′′), 115.6 (C-3′′′, 5′′′), 114.7 (C-3′, 5′), 104.2 (C-10″), 102.1 (C-3″), 101.9 (C-10), 95.7 (C-6), 94.7 (C-8), 94.3 (C-8″), 78.8 (C-2), 42.7 (C-3) see (Supplementary Material Figure S5B); ESIMS: m/z 563.4 for [M+Na]^+^ and 539.3 for [M-H]^−^ see (Supplementary Material Figure S6).

*Methyl gallate* (**3**). White needle crystals; m.p: 189 °C; UV (MeOH) λ_max_: 234 and 275 nm see (Supplementary Material Figure S11); ^1^H-NMR (CD_3_OD, 400 MHz) δ (ppm) 7.05 (s, H-2, 6, 2H), 3.83 (s, OMe-7, 3H) see (Supplementary Material Figure S9A); ^13^C-NMR (CD_3_OD, 100 MHz) δ (ppm) 167.6 (C-7), 145.1 (C-3, 5), 138.3 (C-4), 119.9 (C-1), 108.5 (C-2, 6), 50.8 (7-OCH_3_) see (Supplementary Material Figure S9B); ESIMS: m/z 185.0 for [M+H]^+^ and 183.2 for [M-H]^−^ see (supporting information Figure S10).

*Amentoflavone 4′-O-methyl ether* (**4**). Amorphous yellow powder; UV (MeOH) λ_max_: 234, 270 and 330 nm see (Supplementary Material Figure S14); IR (KBr disc) ν_max_ = 3417, 2922, 2853, 1643, 1616, 1579, 1500, 1432, 1377, 1281, 1248, 1172, 1112, 610, 588, 474, 271, 262, 249, 239 cm^−1^ see (Supplementary Material Figures S15 and S16); ^1^H-NMR (CD_3_OD, 400 MHz) δ (ppm) 8.02 (brd, *J* = 8.8 Hz, H-6′, 1H), 7.96 (d, *J* = 2 Hz, H-2′, 1H), 7.44 (d, *J* = 8.8 Hz, H-2′′′, 6′′′, 2H), 7.26 (d, *J* = 8.8 Hz, H-5′, 1H), 6.72 (d, *J* = 8.8 Hz, H-3′′′, 5′′′, 2H), 6.64 (s, H-3″, 1H), 6.60 (s, H-3, 1H), 6.43 (d, *J* = 2 Hz, H-8, 1H), 6.35 (s, H-6″, 1H), 6.19 (d, *J* = 2 Hz, H-6, 1H), 3.81 (s, OMe-4′, 3H) see (Supplementary Material Figure S12A); ^13^C-NMR (CD_3_OD, 100 MHz) δ (ppm) 182.7 (C-4″), 182.3 (C-4), 164.7 (C-2″), 164.4 (C-7), 164.3 (C-2), 161.9 (C-7″), 161.8 (C-5), 161.2 (C-4′′′), 161.0 (C-4′, C-5″), 158.0 (C-9), 154.8 (C-9″), 130.8 (C-2′), 127.8 (C-2′′′, 6′′′), 127.7 (C-6′), 122.8 (C-1′), 122.0 (C-3′), 121.6 (C-1′′′), 115.4 (C-3′′′, 5′′′), 111.0 (C-5′), 103.9 (C-8″), 103.8 (C-10″, C-10), 103.1 (C-3), 101.8 (C-3″), 98.8 (C-6″), 98.4 (C-6), 93.8 (C-8), 55.0 (4′-OCH_3_) see (Supplementary Material Figure S12B); ESIMS: m/z 575.4 for [M+Na]^+^ and 551.1 for [M-H]^−^ see (Supplementary Material Figure S13).

*Amentoflavone* (**5**). Amorphous yellow powder; UV (MeOH) λmax: 232, 274 and 329 nm see (Supplementary Material Figure S19); IR (KBr disc) ν_max_ = 3417, 2922, 2853, 1651, 1612, 1574, 1493, 1426, 1360, 1285, 1243, 1167, 1106, 1050, 1028, 834, 637, 588, 561, 258 cm^−1^ see (Supplementary Material Figures S20 and S21); ^1^H-NMR (CD_3_OD, 400 MHz) δ (ppm) 7.95 (brs, H-2′, 1H), 7.77 (brd, *J* = 8.4 Hz, H-6′, 1H), 7.46 (d, *J* = 8.8 Hz, H-2′′′, 6′′′, 2H), 7.05 (d, *J* = 8.4 Hz, H-5′, 1H), 6.69 (d, *J* = 8.8 Hz, H-3′′′, 5′′′, 2H), 6.52 (s, H-3″, 1H), 6.52 (s, H-3, 1H), 6.40 (brs, H-8, 1H), 6.32 (s, H-6″, 1H), 6.15 (brs, H-6, 1H) see (Supplementary Material Figure S17A); ^13^C-NMR (CD_3_OD, 100 MHz) δ (ppm) 182.7 (C-4″), 182.2 (C-4), 164.7 (C-2″), 164.5 (C-2), 164.4 (C-7), 162.5 (C-7″), 161.7 (C-5), 161.0 (C-4′′′, C-5″), 159.6 (C-4′), 157.8 (C-9), 154.9 (C-9″), 131.3 (C-2′), 127.8 (C-2′′′, 6′′′), 127.4 (C-6′), 121.7 (C-1′′′), 121.6 (C-1′), 120.2 (C-3′), 116.0 (C-5′), 115.4 (C-3′′′, 5′′′), 103.9 (C-8″), 103.8 (C-10, C-10″), 102.4 (C-3), 101.8 (C-3″), 98.8 (C-6″), 98.7 (C-6), 93.7 (C-8) see (Supplementary Material Figure S17B); ESIMS: m/z 561.4 for [M+Na]^+^ and 537.1 for [M-H]^−^ see (Supplementary Material Figure S18).

*Naringenin 7-O- β-D glucoside* (**6**)**.** Amorphous light brown powder; UV (MeOH) λ_max_: 243, 294 and 326 nm see (Supplementary Material Figure S25); ^1^H-NMR (CD_3_OD, 400 MHz) δ (ppm) 7.21 (d, *J* = 8.4 Hz, H-2′, 6′, 2H), 6.71 (d, *J* = 8.4 Hz, H-3′, 5′, 2H), 6.10 (brs, H-8, 1H), 6.08 (brs, H-6, 1H), 5.27 (brd, *J* = 12.4 Hz, H-2, 1H), 3.06 (dd, *J* = 12.8, 16.8 Hz, H-3*_ax_*, 1H), 2.64 (brd, *J* = 16.8 Hz, H-3*_equ_*, 1H), 4.86 (d, *J* = 8 Hz, Glu H-1, 1H), 3.75 (m, Glu H-6*_equ_*, 1H). 3.58 (m, Glu H-6*_ax_*, 1H), 3.28-3.36 (m, Glu H-2–Glu H-5, 4H) see (Supplementary Material Figure S22A); DEPTQ-NMR (CD_3_OD, 100 MHz) δ (ppm) 197.1 (C-4), 165.6 (C-7), 163.5 (C-5), 163.2 (C-9), 157.7 (C-4′), 129.4 (C-1′), 127.7 (C-2′, 6′), 114.9 (C-3′, 5′), 103.4 (C-10), 96.5 (C-6), 95.4 (C-8), 79.3 (C-2), 42.7 (C-3), 99.8 (Glu C-1), 76.8 (Glu C-5), 76.3 (Glu C-3), 73.2 (Glu C-2), 69.6 (Glu C-4), 60.8 (Glu C-6) see (Supplementary Material Figure S22B); HSQC and HMBC NMR (CD_3_OD) see (Supplementary Material Figure S23); ESIMS: m/z 457.1 for [M+Na]^+^ and 433.1 for [M-H]^−^ see (Supplementary Material Figure S24).

*4′,4′′′Biapigenin-8″-C-β-D-glucopyranosyl [1′′′′′′→3′′′′′]β-D-glucopyranoside-8-C-β-D-glucopyranoside)* (**7**)**.** Amorphous light brown powder; ${\left[\alpha \right]}_{D}^{20}$ +36.45 (C 0.1, methanol); UV (MeOH) λ_max_: 238, 268 and 324 nm see (Supplementary Material Figure S29); ^1^H-NMR (CD_3_OD, 400 MHz) δ (ppm) 7.99 (d, *J* = 8 Hz, H-2′′′, 6′′′, 2H), 7.85 (d, *J* = 8 Hz, H-2′, 6′, 2H), 6.95 (d, *J* = 8 Hz, H-3′, 5′, 3′′′, 5′′′, 4H), 6.62 (brs, H-3, 3″, 2H), 6.27 (brs, H-6, 6″, 2H), 5.14 (d, *J* = 9.2 Hz, Glu-I-H-1, 1H), 3.83-3.98 (m, Glu-I-H-6, 2H), 3.59 (m, Glu-I-H-4, 1H), 3.48-3.56 (m, Glu-I-H-5, 1H), 3.01 (m, Glu-I-H-2, 1H), 2.93 (m, Glu-I-H-3, 1H), (brs, H-3, 3″, 2H), 5.07 (d, *J* = 9.6 Hz, Glu-II-H-1, 1H), 4.41 (m, Glu-II-H-5, 1H), 4.34 (m, Glu-II-H-3, 1H), 3.37-3.42 (m, Glu-II-H-6, 2H), 3.17 (m, Glu-II-H-2, 1H), 3.10-3.17 (m, Glu-II-H-4, 1H), 4.21 (d, *J* = 7.6 Hz, Glu-III-H-1, 1H), 3.76 (m, Glu-III-H-5, 1H), 3.71 (m, Glu-III-H-4, 1H), 3.30 (m, Glu-III-H-6, 2H). 3.24 (m, Glu-III-H-3, 1H), 2.73 (m, Glu-III-H-2, 1H) see (Supplementary Material Figure S26A); DEPTQ-NMR (CD_3_OD, 100 MHz) δ (ppm) 182.8 (C-4″), 182.7 (C-4), 165.1 (C-2″), 164.8 (C-2), 163.5 (C-7″), 163.2 (C-7), 161.5 (C-5″), 161.4 (C-4′, C-4′′′), 161.2 (C-5), 156.8 (C-9″), 156.2 (C-9), 128.7 (C-2′′′, 6′′′), 128.2 (C-2′, 6′), 122.1 (C-1′, C-1′′′), 115.7 (C-3′′′, 5′′′), 115.6 (C-3′, 5′), 104.1 (C-10″), 104.0 (C-10), 103.6 (C-8″), 103.0 (C-8), 102.5 (C-3″), 102.3 (C-3), 99.3 (C-6″), 98.1 (C-6), 81.4 (Glu-I-C-5), 76.1 (Glu-I-C-3), 74.3 (Glu-I-C-2), 73.3 (Glu-I-C-1), 69.8 (Glu-I-C-4), 61.5 (Glu-I-C-6), 81.0 (Glu-II-C-5), 80.2 (Glu-II-C-3), 76.2 (Glu-II-C-2), 72.2 (Glu-II-C-1), 69.6 (Glu-II-C-4), 61.1 (Glu-II-C-6), 104.3 (Glu-III-C-1), 78.7 (Glu-III-C-5), 76.4 (Glu-III-C-3), 75.6 (Glu-III-C-2), 70.6 (Glu-III-C-4), 60.8 (Glu-III-C-6) see (Supplementary Material Figure S26B); HSQC and HMBC NMR (CD_3_OD) see (Supplementary Material Figure S27); HRMS (ESI) m/z 1009.26259 [M+H]^+^ see (Supplementary Material Figure S28).

### 3.4. Biological Activity

#### 3.4.1. Toxoplasmocidal Activity

The assay was carried out using virulent RH *T*. *gondii* strain, which was obtained from the Medical Parasitology Department, Faculty of Medicine, Alexandria University, Egypt. Different concentrations of the total methanol extract of the *C*. *rumphii* leaves and its different fractions were tested for toxplasmocidal activity according to the method reported by [31]. The mean effective concentration (EC_50_) in μg/mL was calculated and compared to that of cotrimoxazole as a reference drug.

#### 3.4.2. Cytotoxic Activity

Hepatocellular carcinoma (HEPG-2), mammary gland breast carcinoma (MCF-7), colorectal carcinoma (HCT-116), prostate carcinoma (PC-3), cervical carcinoma (HELA) and the normal (WISH) amniotic cell lines were obtained from the American Type Culture Collection (ATCC) via Holding Company for Biological Production and Vaccines (VACSERA), Cairo-Egypt**.**

The cytotoxicity assay was carried out according to the reported procedures using the MTT assay method [32,33]. The assay was carried out using seven different concentrations (1.56, 3.125, 6.25, 12.5, 25, 50 and 100 μg/mL) of the total methanol extract of *C*. *rumphii* in DMSO against the tested cancer cell lines and one normal cell line (WISH) to ensure the safety of the plant extract on normal cells. The most affected cancer cell lines were tested against the extracts of petroleum ether, methylene chloride, ethyl acetate and *n*-butanol fractions and the constituents of the most active fraction were also tested using the same previous concentrations. Doxorubicin was used as a positive control. IC_50_ was calculated and the cytotoxic effect was assessed according to the classification of Hossan and Abu Melha, 2014 [13].

### 3.5. Statistical Analysis

All experiments were carried out at least three times, and the data were expressed as the mean ± standard error of the mean (SEM).

## 4. Conclusions

In conclusion, the biological screening for *C. rumphii* different fractions indicated that the ethyl acetate fraction has potent toxoplasmocidal and cytotoxic activities against various cell lines. Therefore, the phytochemical investigation of the most active fraction of the *C. rumphii* leaves was carried out and resulted in the isolation of a previously undescribed biflavonoid C-glycoside in addition to six known compounds, three of them were isolated for the first time from *C. rumphii* leaves. These compounds were identified using different spectroscopic techniques and compared to the data reported in the literature. In addition, the isolated compounds were evaluated for cytotoxic activity using the MTT assay method. The results showed that compounds (**3**, **6** and **7**) have significant cytotoxic activity. Future studies for in vivo assessment of these activities are necessary.

## Data Availability

All data are included in the text and the supporting material file.

## Supplementary Materials

The following supporting information can be downloaded at: https://www.mdpi.com/article/10.3390/plants11212867/s1, Table S1: Toxoplasmocidal effect of *C. rumphii* methanol extract and its different fractions against *T. gondii*; Table S2: Cytotoxic effect of *C. rumphii* methanol extract against different cell lines; Table S3: Cytotoxic effect of *C. rumphii* different fractions against HEPG-2, HCT-116 and HELA cell lines; Table S4: Cytotoxic effect and selectivity index of *C. rumphii* ethyl acetate fraction and its constituents against HEPG-2, HCT-116 and HELA cell lines; Figure S1: (A) ^1^H and (B) ^13^C-NMR spectrum of compound (**1**) (CD_3_OD); Figure S2: (A) HSQC and (B) HMBC spectrums of compound (**1**) (CD_3_OD); Figure S3: ESIMS “positive (A) and negative modes (B)” of compound (**1**); Figure S4: UV spectrum of compound (**1**) in CH_3_OH; Figure S5: (A) ^1^H and (B) ^13^C-NMR spectrum of compound (**2**) (CD_3_OD); Figure S6: ESIMS “positive (A) and negative modes (B) of compound (**2**); Figure S7: UV spectrum of compound (**2**) in CH_3_OH; Figure S8: IR spectrum of compound (**2**) in KBr disc; Figure S9: (A) ^1^H and (B) ^13^C NMR spectrum of compound (**3**) (CD_3_OD); Figure S10: ESIMS “positive (A) and negative modes (B)” of compound (**3**); Figure S11: UV spectrum of compound (**3**) in CH_3_OH; Figure S12: (A) ^1^H and (B) ^13^C-NMR spectrum of compound (**4**) (CD_3_OD); Figure S13: ESIMS “positive (A) and negative modes (B)” of compound (**4**); Figure S14: UV spectrum of compound (**4**) in CH_3_OH; Figure S15: IR spectrum of compound (**4**) in KBr disc; Figure S16**.** IR fingerprint spectrum of compound (**4**) and bilobetin authentic sample in KBr disc; Figure S17: (A) ^1^H and (B) ^13^C-NMR spectrum of compound (**5**) (CD_3_OD); Figure S18: ESIMS “positive (A) and negative modes (B)” of compound (**5**); Figure S19: UV spectrum of compound (**5**) in CH_3_OH; Figure S20: IR spectrum of compound (**5**) in KBr disc; Figure S21. IR fingerprint spectrum of compound (**5**) and amentoflavone authentic sample in KBr disc; Figure S22: (A) ^1^H and (B) DEPTQ NMR spectrum of compound (**6**) (CD_3_OD); Figure S23: (A) HSQC and (B) HMBC spectrums of compound (**6**) (CD_3_OD); Figure S24: ESIMS “positive (A) and negative modes (B)” of compound 6; Figure S25: UV spectrum of compound (**6**) in CH_3_OH; Figure S26: (A) ^1^H and (B) DEPTQ NMR spectrum of compound (**7**) (CD_3_OD); Figure S27: (A) HSQC and (B) HMBC spectrums of compound (**7**) (CD_3_OD); Figure S28: HRMS (ESI^+^) (positive mode) of compound (**7**) with zooming to molecular ion region; Figure S29: UV spectrum of compound (**7**) in CH_3_OH
