# Supplementary material for: Toxoplasmocidal and Cytotoxic Activities Guided Isolation and Characterization of an Undescribed Bioflavonoid-di-C-glucoside from Cycas rumphii Miq. Cultivated in Egypt
Source: Plants (Basel). 2022 Oct 27;11(21):2867. doi: 10.3390/plants11212867 (PMC9655732; doi:10.3390/plants11212867)
Supplement: Supplementary file 1 [file plants-11-02867-s001.zip › plant-1953480 supplementary.pdf]

plants

MDPI

Article

Toxoplasmocidal and Cytotoxic Activities Guided Isolation and Characterization of an Undescribed Bioflavonoid-di-*C* -glucoside from *Cycas rumphii* Miq. Cultivated in Egypt

Supplementary Materials

Drug	EC_{50} ± SEM (µg/mL)
Cotrimoxazole	4.18±0.3
C. rumphii MeOH extract	5.15±0.3
Pet. ether fraction	41.27±1.7
Methylene chloride fraction	23.01±0.9
Ethyl acetate fraction	3.51±0.2
n-butanol fraction	5.85±0.3

Table S1. Toxoplasmocidal effect of *C. rumphii* methanol extract and its different fractions against *T. gondii*

Drug	*In vitro* Cytotoxicity IC_{50} ± SEM (µg/mL)					
	HEPG-2	MCF-7	HCT-116	PC3	WISH	HELA
Doxorubicin	4.50±0.2	4.17±0.2	5.23±0.3	8.87±0.6	7.79±0.5	5.57±0.4
Total MeOH extract of *C. rumphii*	10.09±0.9	17.17±1.4	12.58±1.1	23.54±1.8	53.72±1.9	11.79±1.0

Table S2. Cytotoxic effect of *C. rumphii* methanol extract against different cell lines

Drug	*In vitro* Cytotoxicity IC_{50} ± SEM (μg/mL)			
	HEPG-2	HCT-116	HELA	WISH
Doxorubicin	4.50±0.2	5.23±0.3	5.57±0.4	7.79±0.5
Pet. ether fraction	42.01±3.3	38.37±3.0	46.99±3.5	96.69±4.17
Methylene chloride fraction	24.56±2.1	29.39±2.4	19.04±1.4	86.75±2.2
Ethyl acetate fraction	6.98±0.5	8.70±0.9	7.94±0.8	36.17±0.9
n-butanol fraction	8.13±0.7	13.68±1.2	9.48±1.0	43.19±1.5

Table S3. Cytotoxic effect of *C. rumphii* different fractions against HEPG-2, HCT-116 and HELA cell lines

Drug	*In vitro* Cytotoxicity IC_{50} ± SEM (μg/mL)				Selectivity index		
	HEPG-2	HCT-116	HELA	WISH	HEPG-2	HCT-116	HELA
Doxorubicin	4.50±0.2	5.23±0.3	5.57±0.4	7.79±0.5	1.73	1.48	1.39
Ethyl acetate fraction	6.98±0.5	8.70±0.9	7.94±0.8	36.17±0.9	5.18	4.15	4.55
Compound 1	86.19±4.2	65.35±3.5	72.86±3.8	41.83±2.7	0.48	0.64	0.57
Compound 2	34.50±2.2	27.66±2.1	31.17±2.3	81.72±4.3	2.36	2.95	2.62
Compound 3	8.67±0.6	6.24±0.5	10.08±0.9	45.90±3.0	5.29	7.35	4.55
Compound 4	67.39±3.4	42.03±2.6	54.74±3.3	100±3.7	1.48	2.37	1.82
Compound 5	50.41±2.9	36.69±2.4	47.20±2.7	74.26±3.6	1.47	2.02	1.57
Compound 6	14.49±1.1	11.63±0.9	23.32±2.0	38.60±2.4	2.66	3.31	1.65
Compound 7	21.47±1.8	18.17±1.4	15.66±1.3	76.66±3.9	3.57	4.21	4.89

Table S4. Cytotoxic effect and selectivity index of *C. rumphii* ethyl acetate fraction and its constituents against HEPG-2, HCT-116 and HELA cell lines

(A)

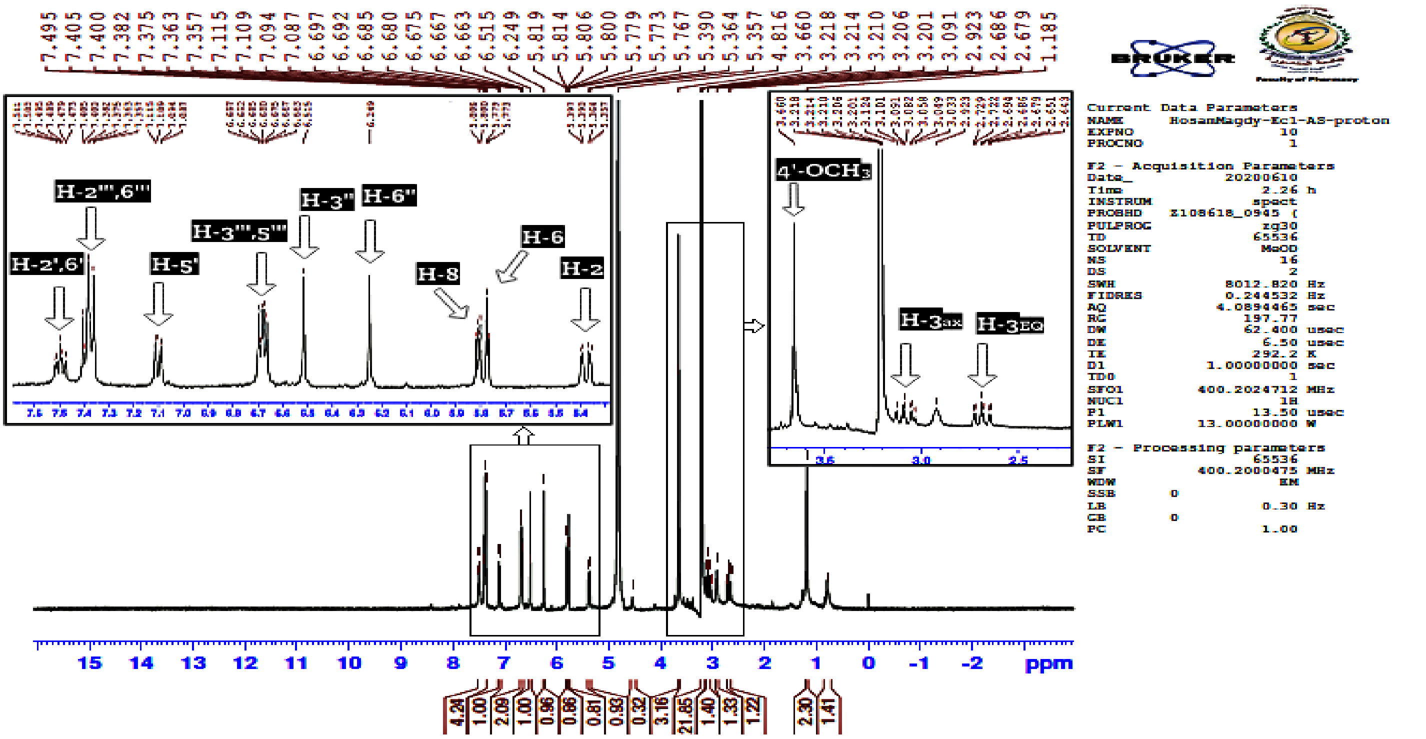

(B)

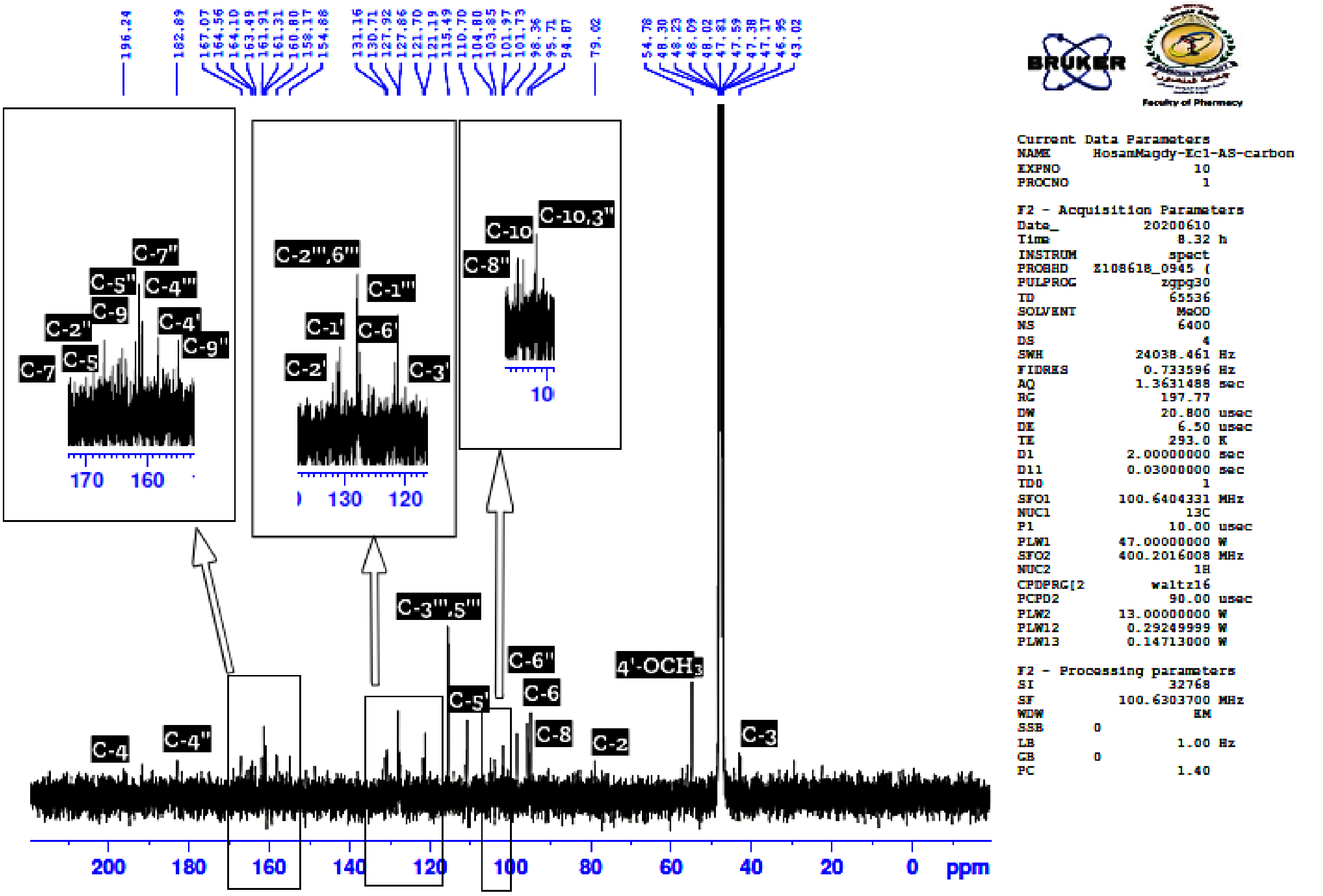

Figure S1. (A) ^{1}H and (B) ^{13}C-NMR spectrum of compound (**1**) (CD_3OD)

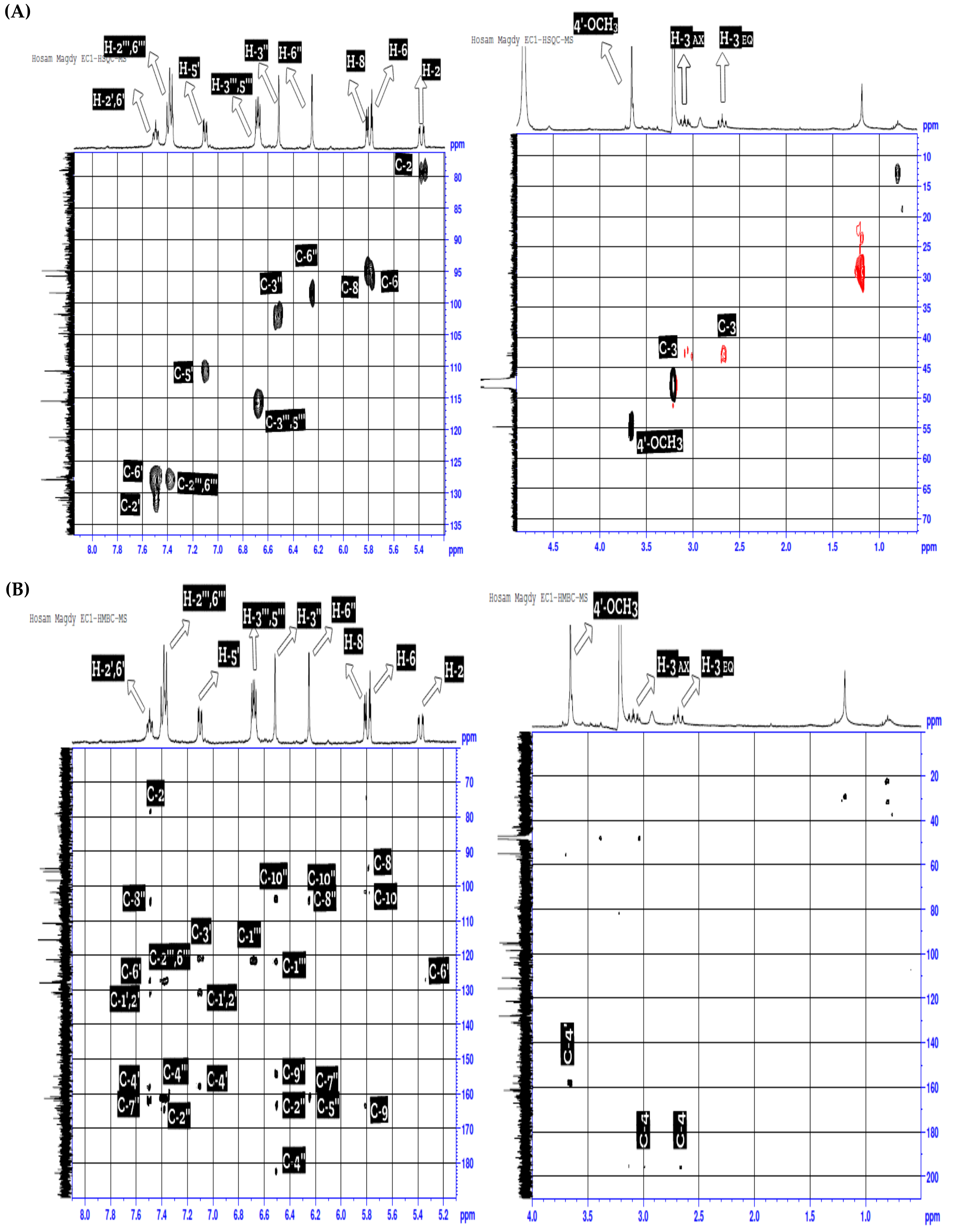

Figure S2: (A) HSQC and (B) HMBC spectrums of compound (**1**) (CD_3OD)

(A)

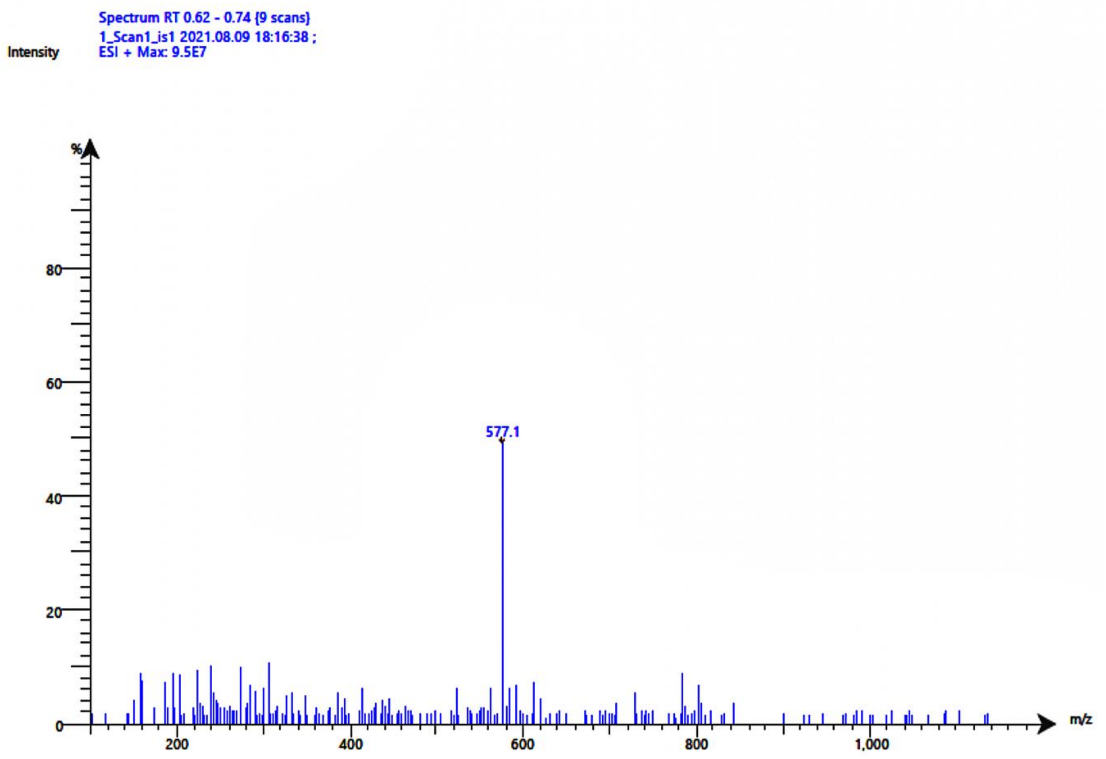

(B)

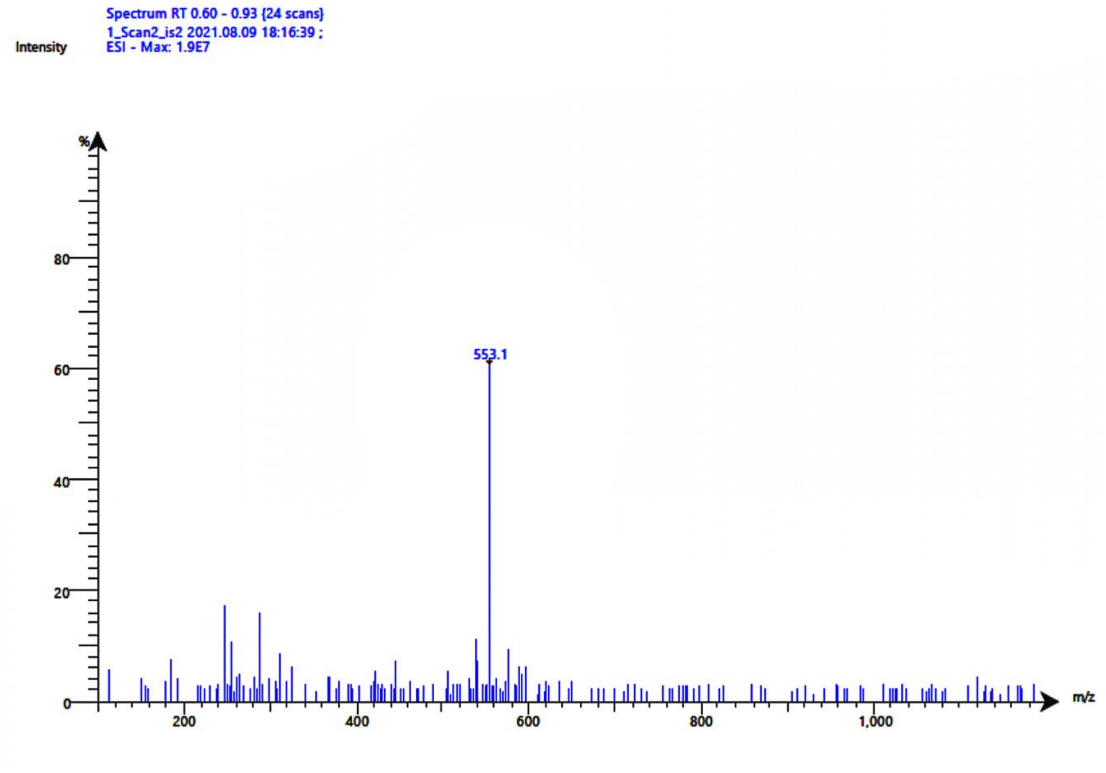

Figure S3. ESIMS "positive (A) and negative modes (B)" of compound (**1**)

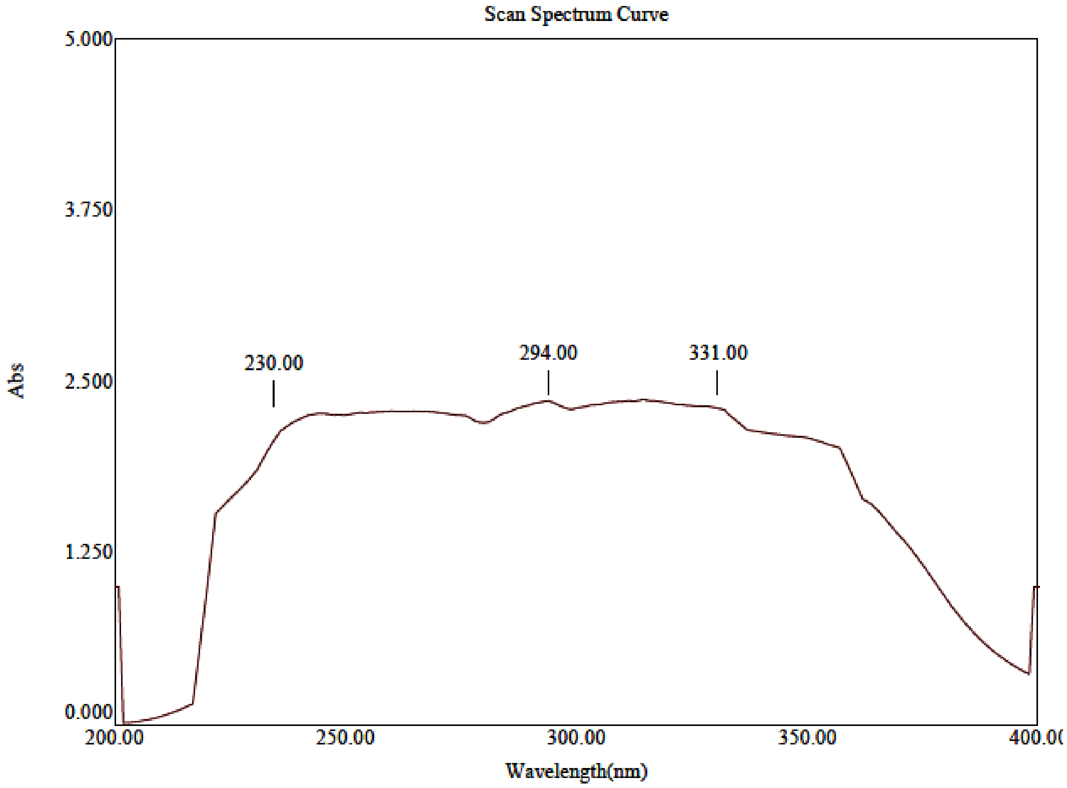

Figure S4. UV spectrum of compound (**1**) in CH_3OH

(A)

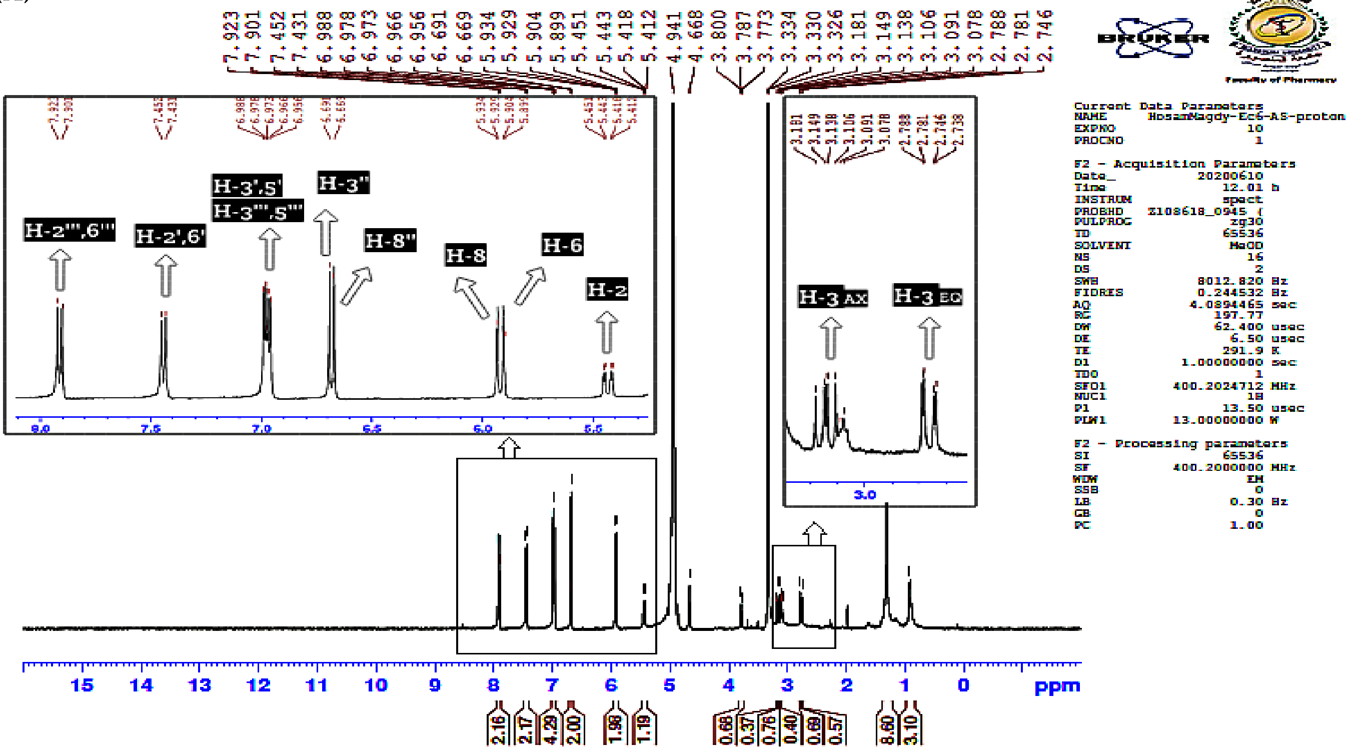

(B)

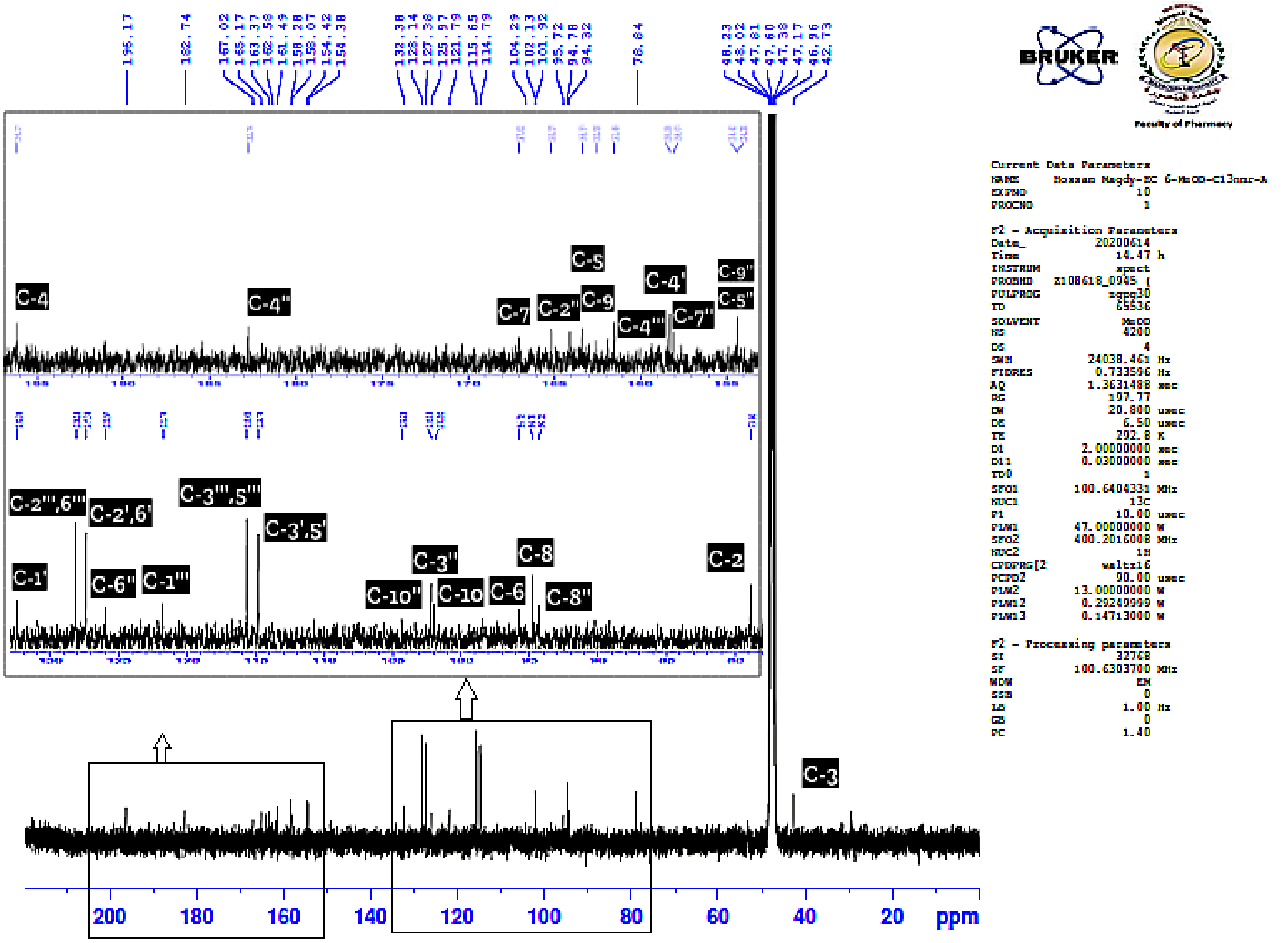

Figure S5. (A) ^{1}H and (B) ^{13}C-NMR spectrum of compound (**2**) (CD_3OD)

(A)

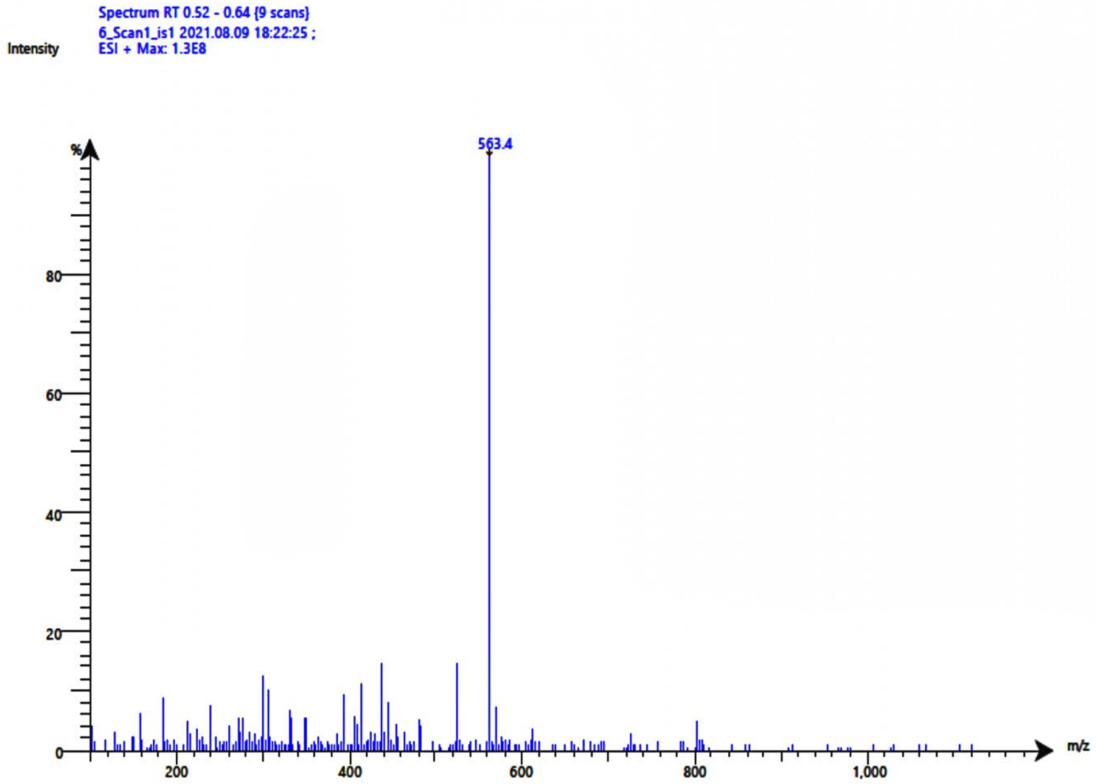

(B)

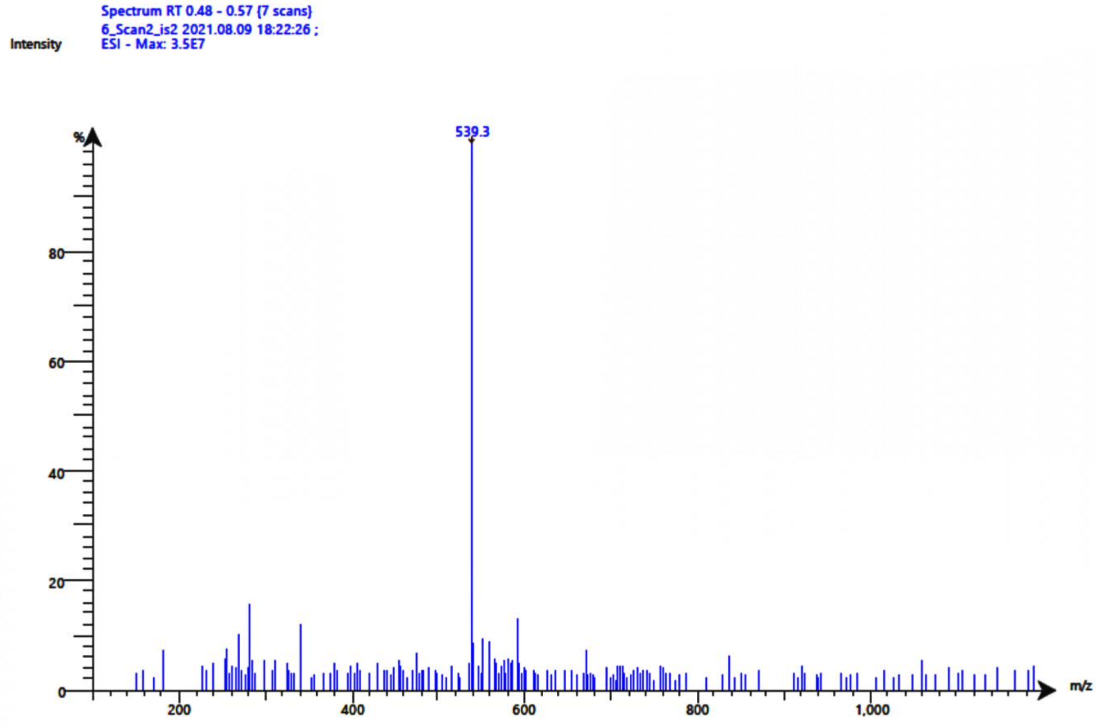

Figure S6. ESIMS ''positive (A) and negative modes (B) of compound (**2**)

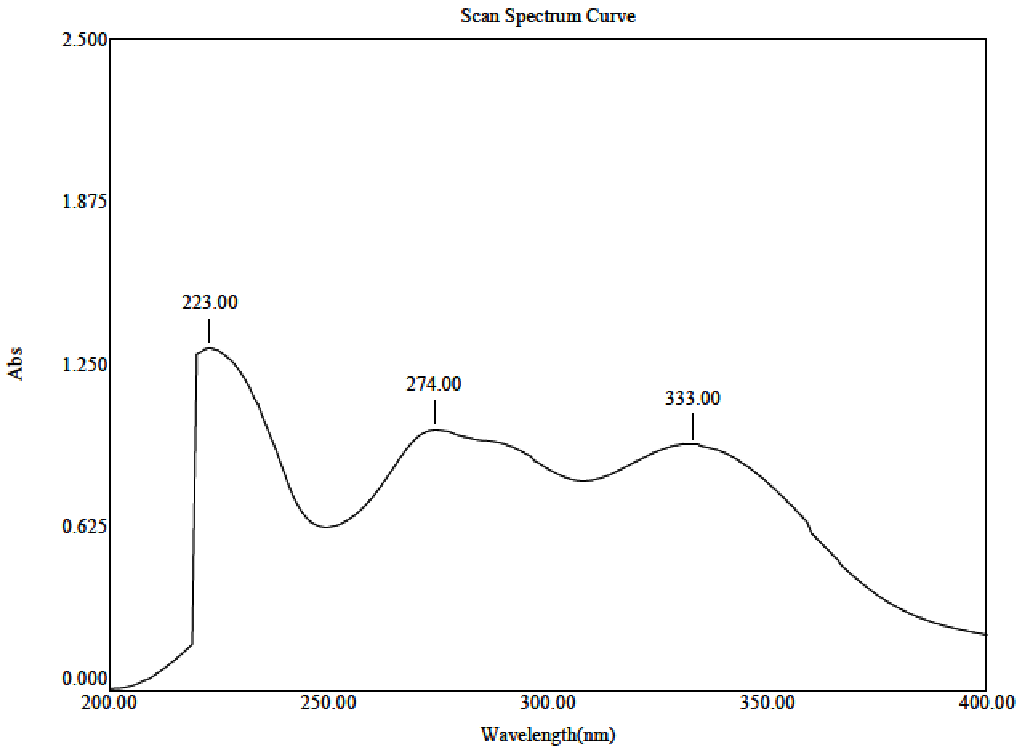

Figure S7. UV spectrum of compound (**2**) in CH_3OH

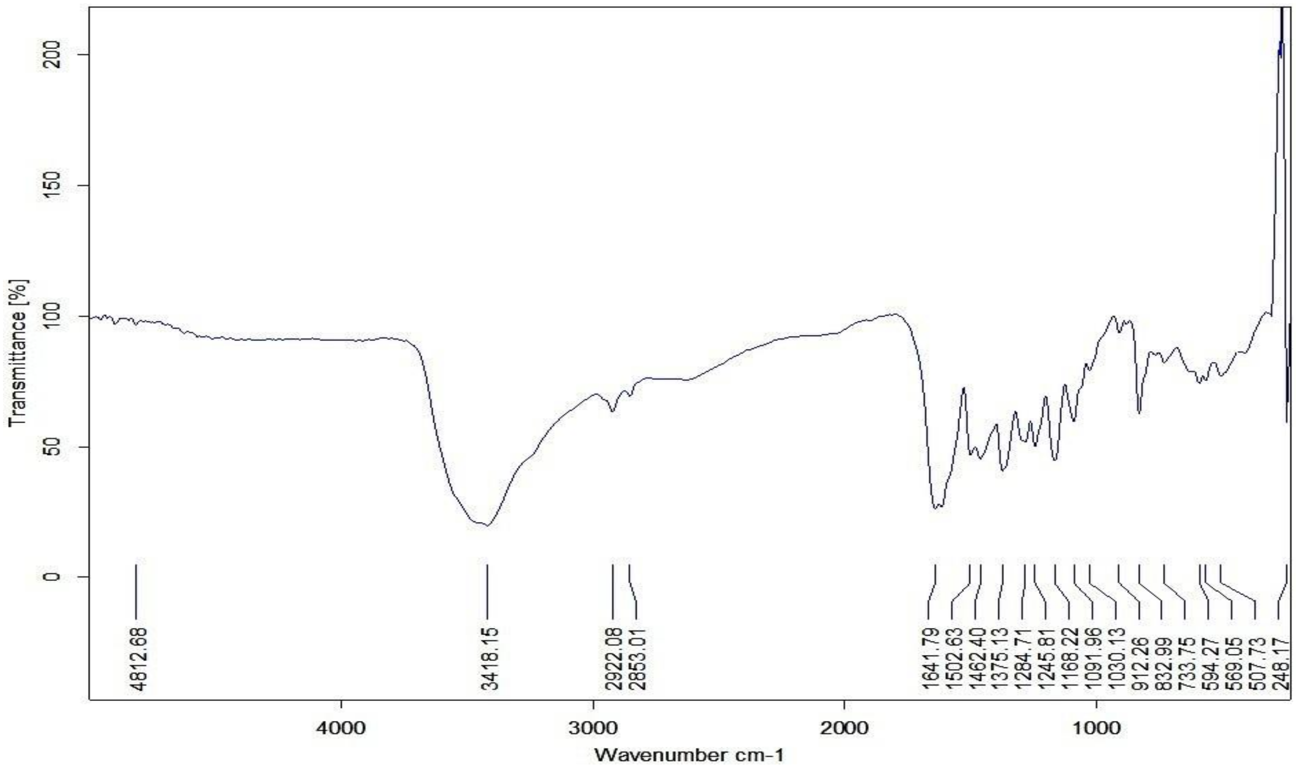

Figure S8. IR spectrum of compound (**2**) in KBr disc

(A)

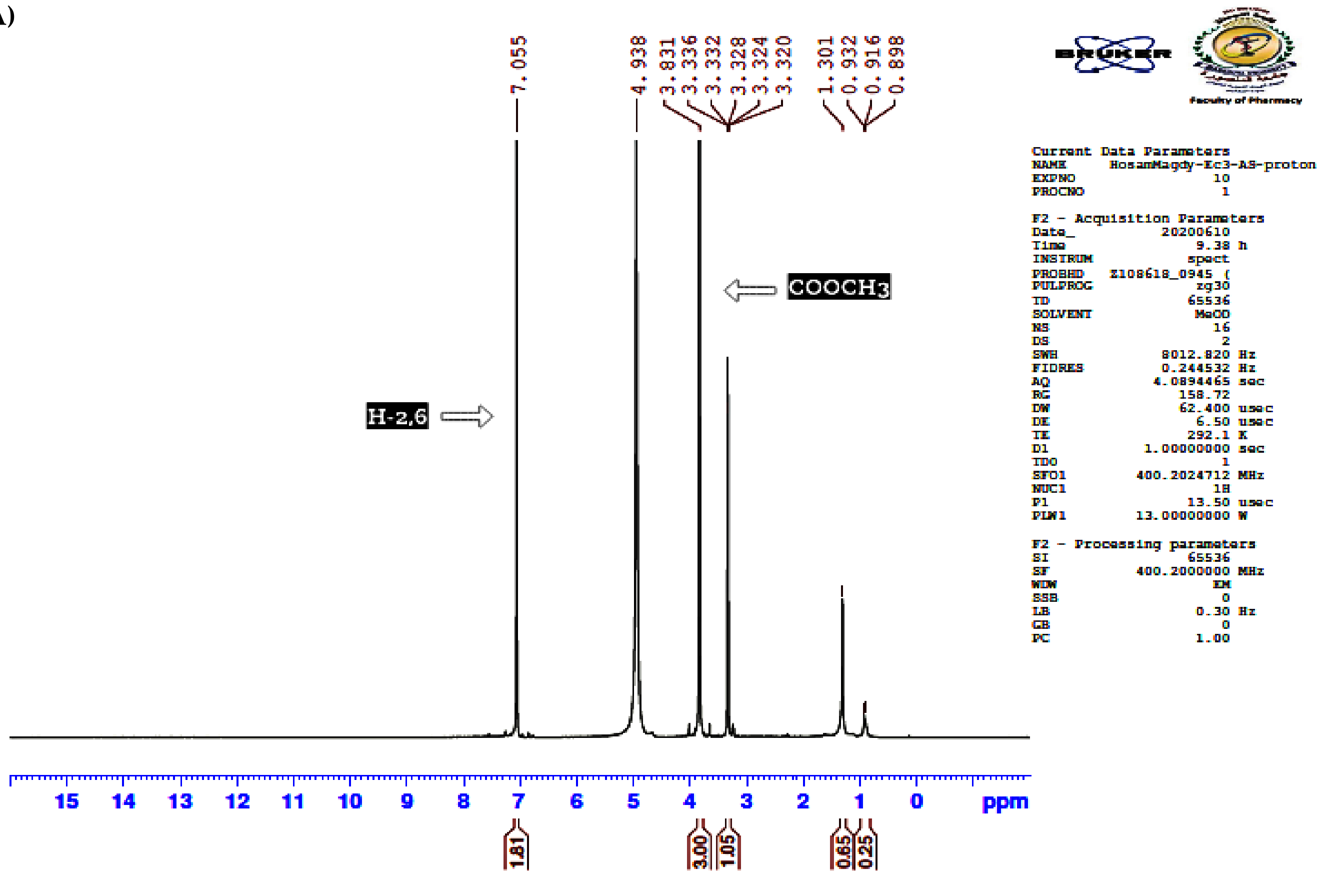

(B)

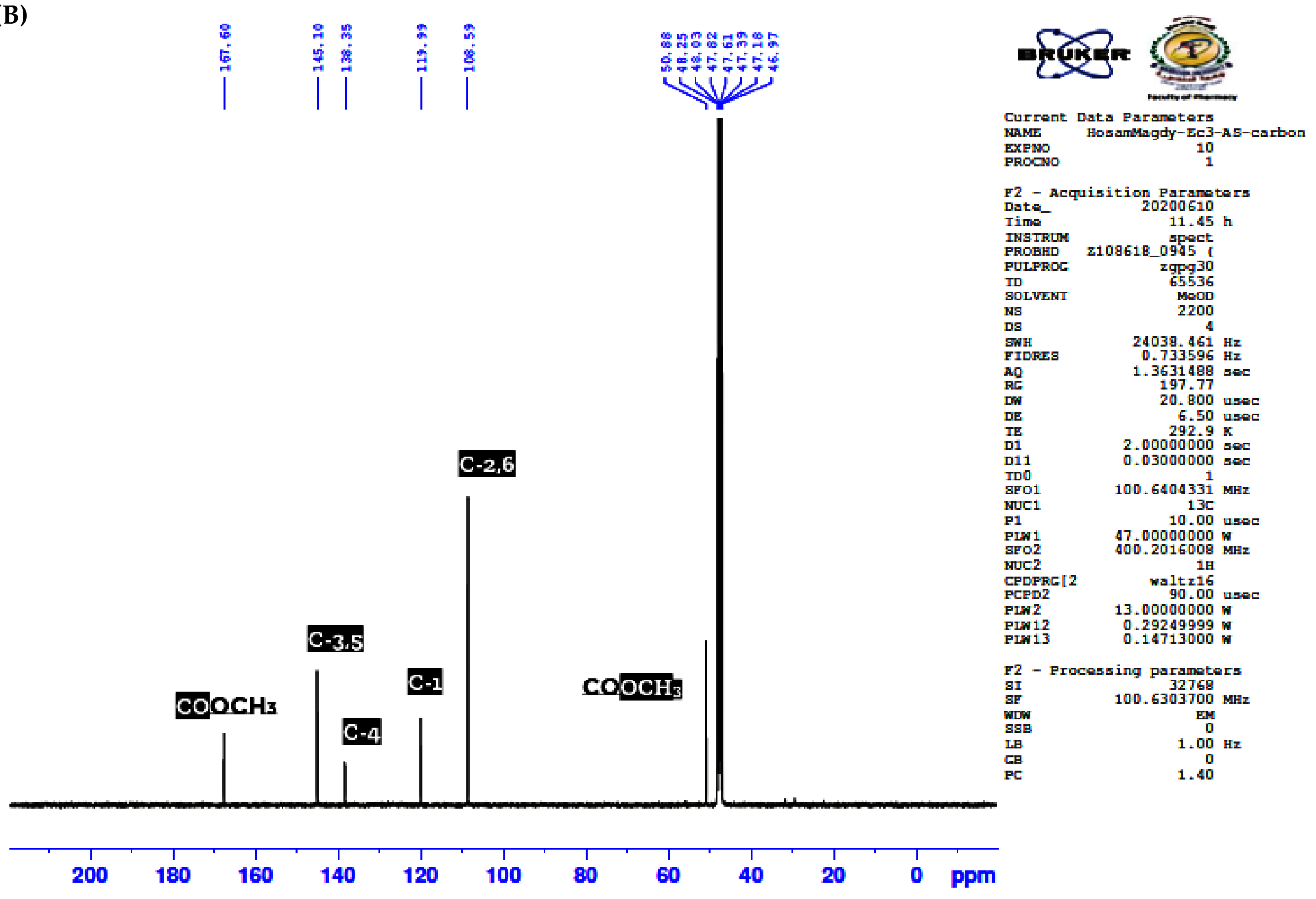

Figure S9. (A) ^{1}H and (B) ^{13}C NMR spectrum of compound (**3**) (CD_3OD)

(A)

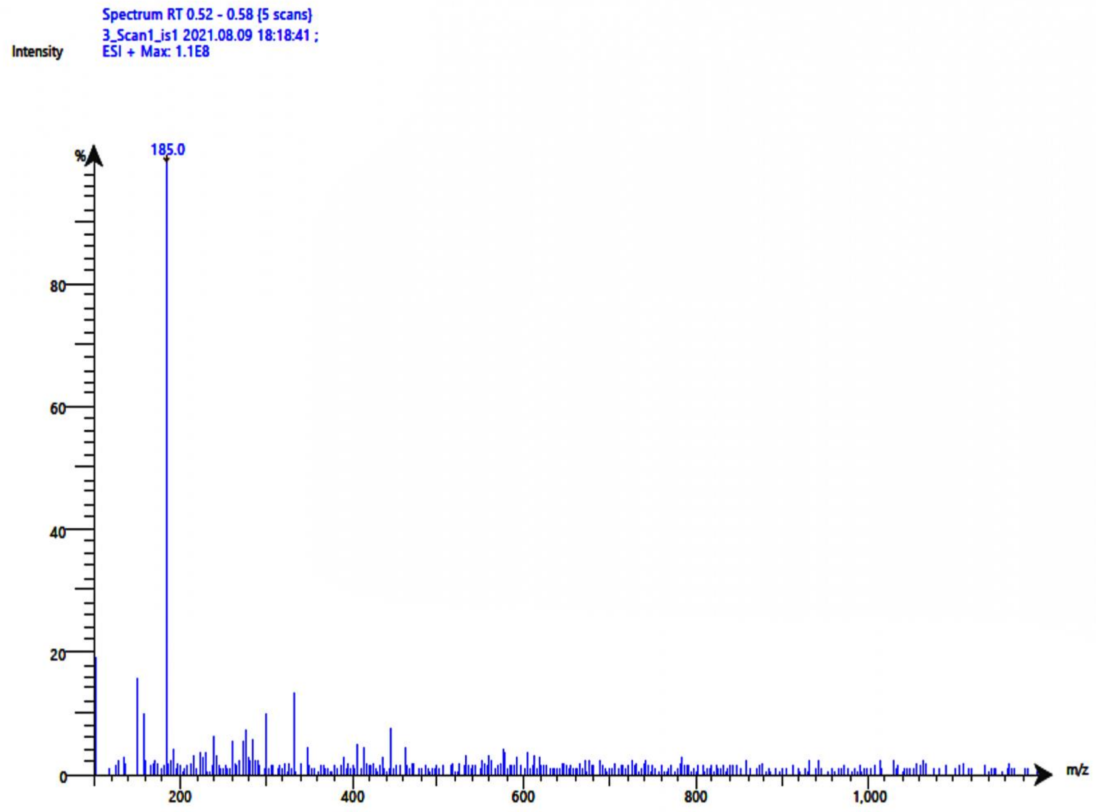

(B)

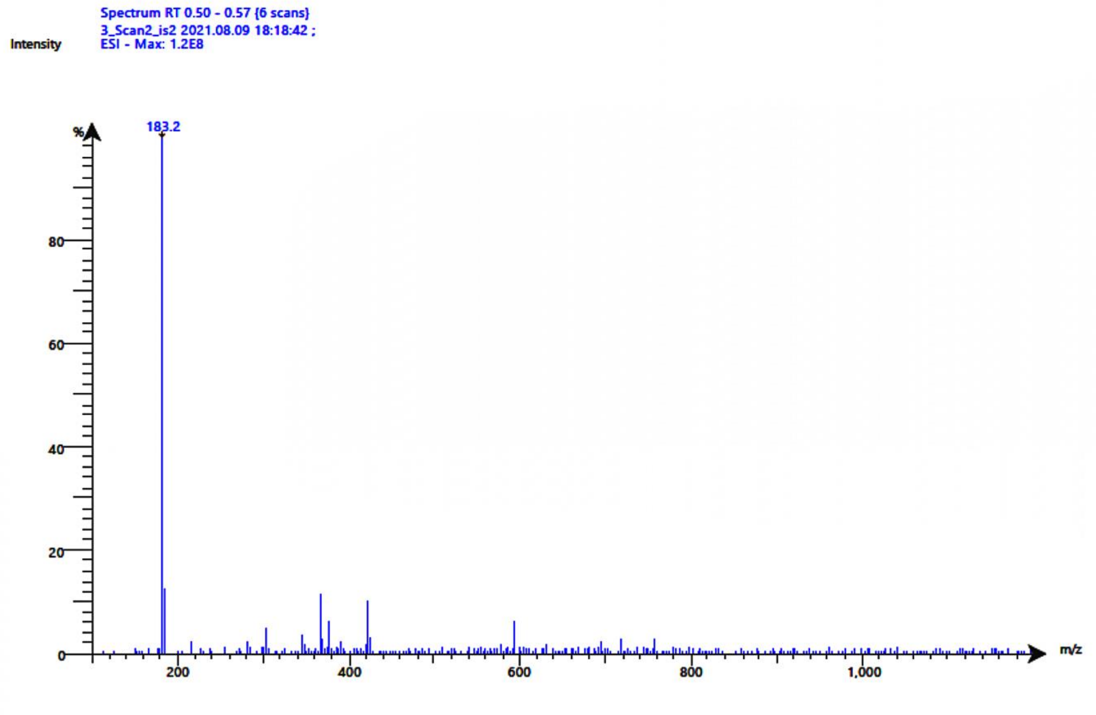

Figure S10. ESIMS "positive (A) and negative modes (B)" of compound (**3**)

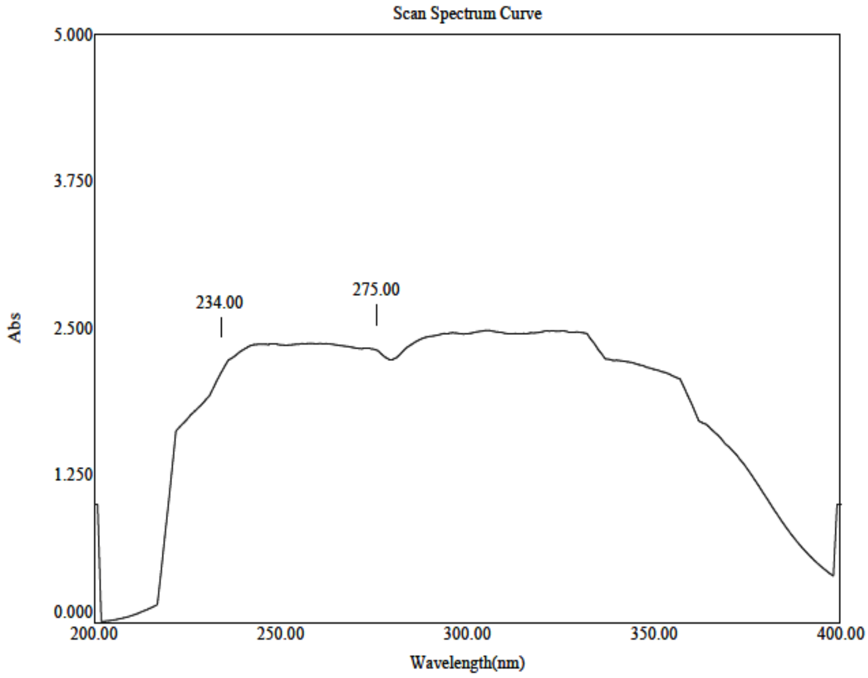

Figure S11. UV spectrum of compound (**3**) in CH_3OH

(A)

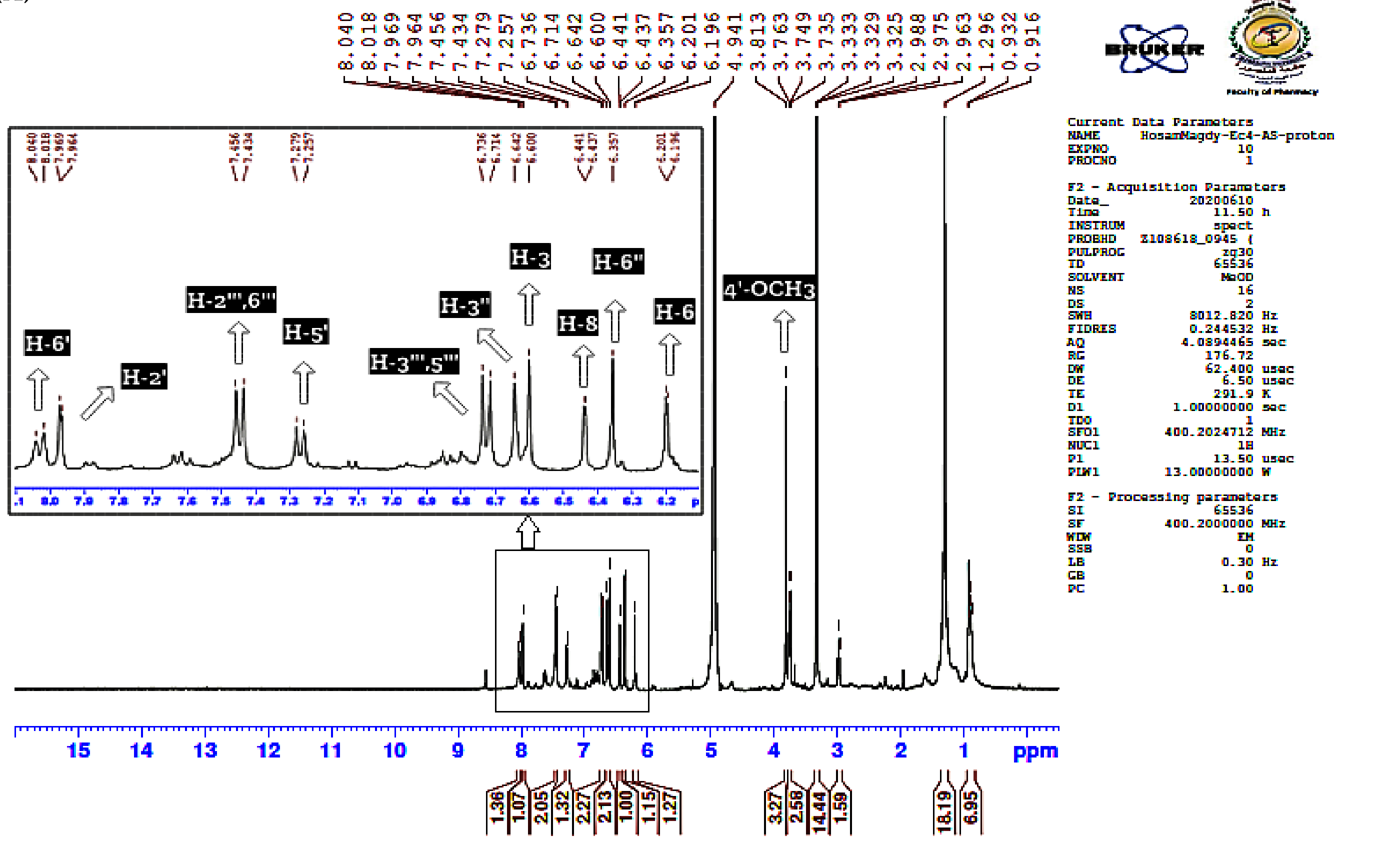

(B)

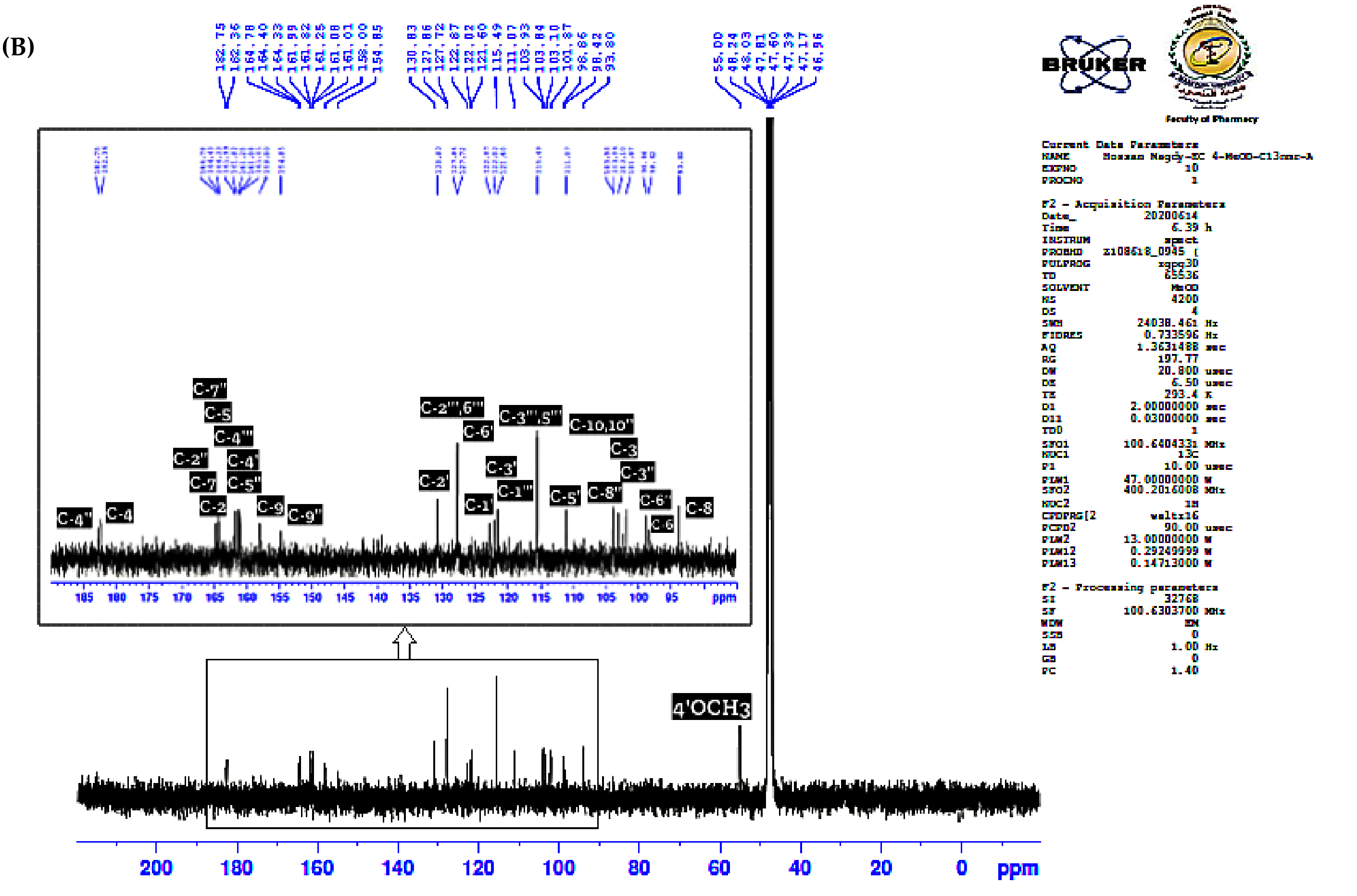

Figure S12. (A) ^{1}H and (B) ^{13}C-NMR spectrum of compound (**4**) (CD_3OD)

(A)

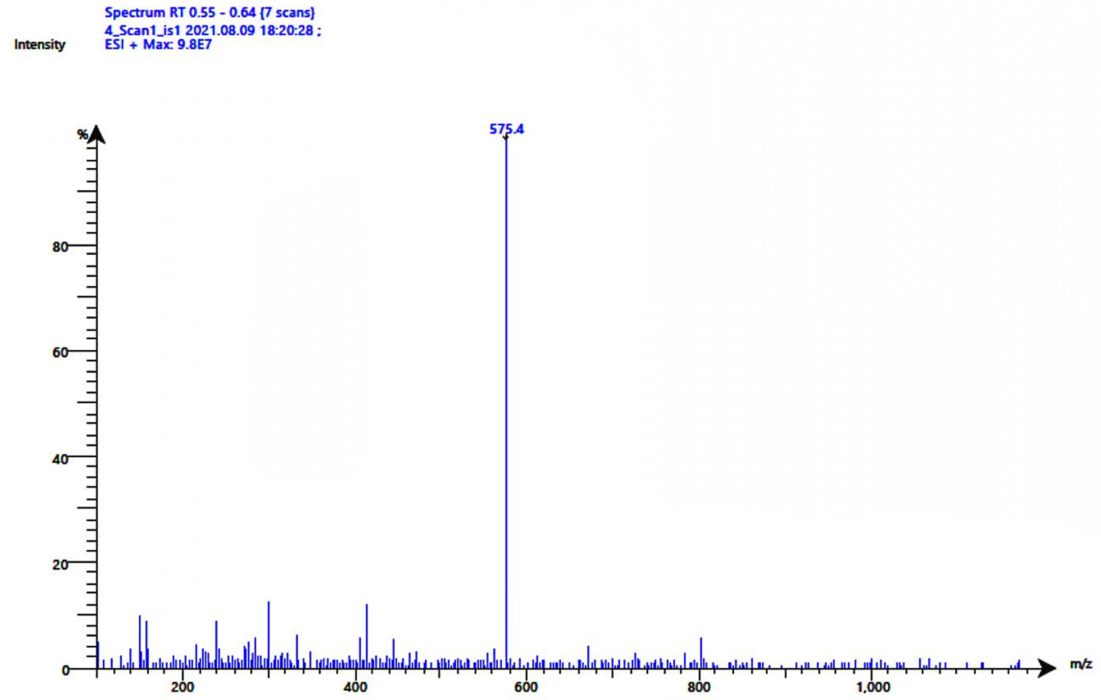

(B)

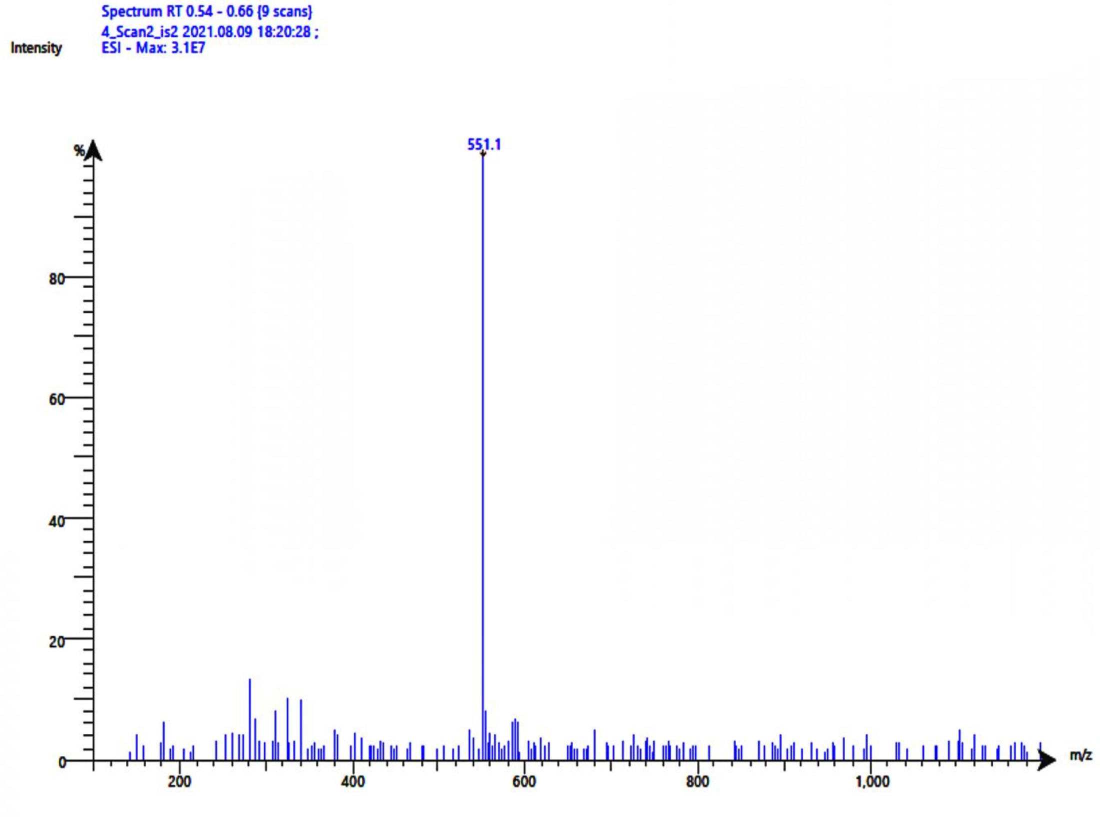

Figure S13. ESIMS "positive (A) and negative modes (B)" of compound (**4**)

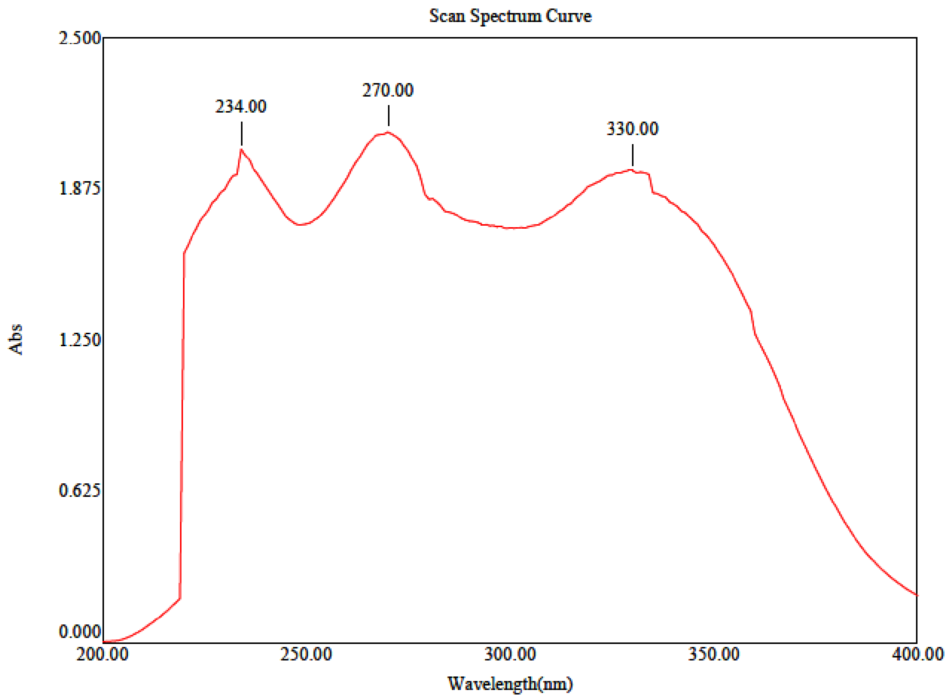

Figure S14. UV spectrum of compound (**4**) in CH_3OH

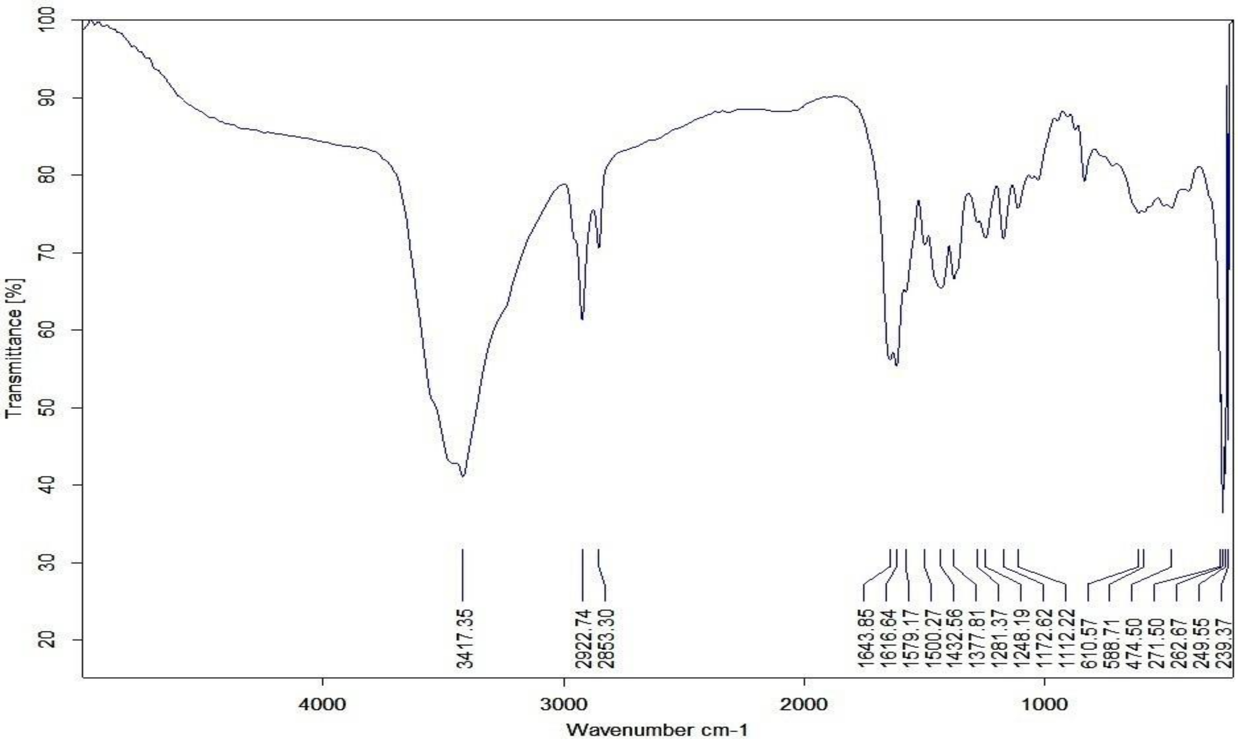

Figure S15. IR spectrum of compound (**4**) in KBr disc

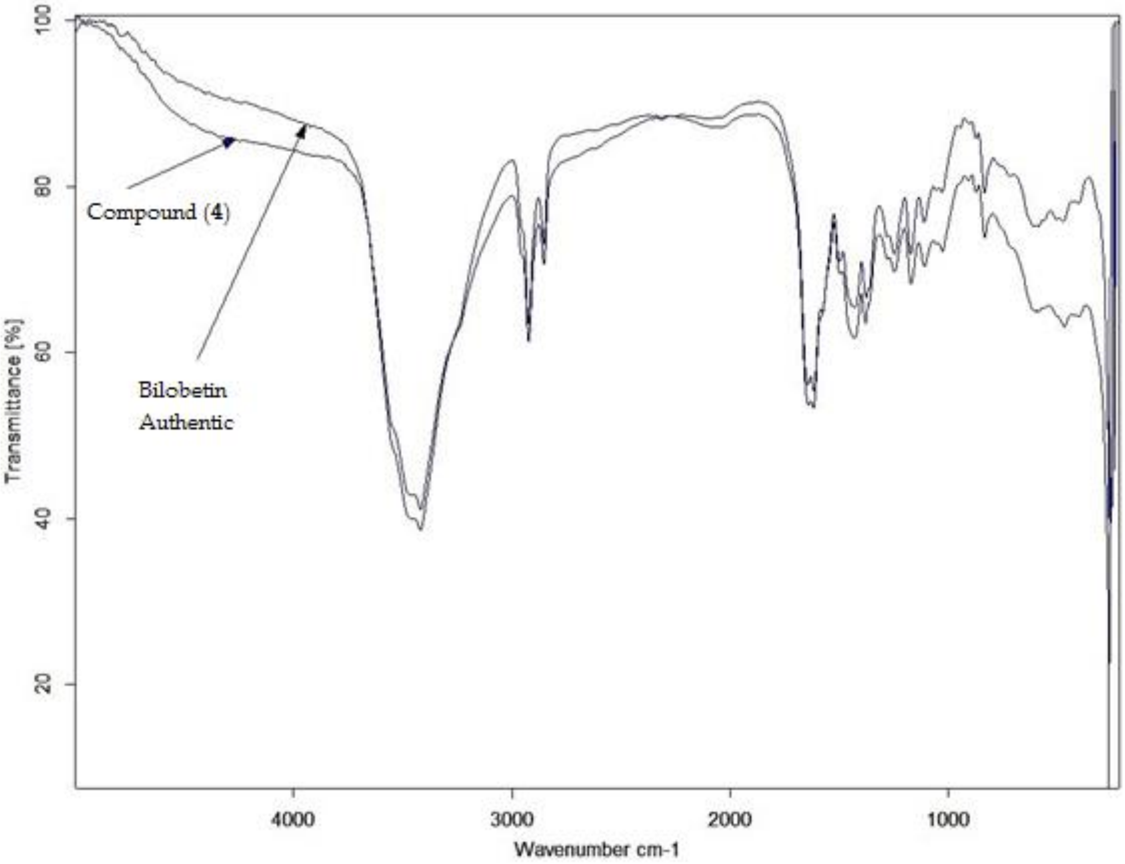

Figure S16. IR fingerprint spectrum of compound (**4**) and bilobetin authentic sample in KBr disc

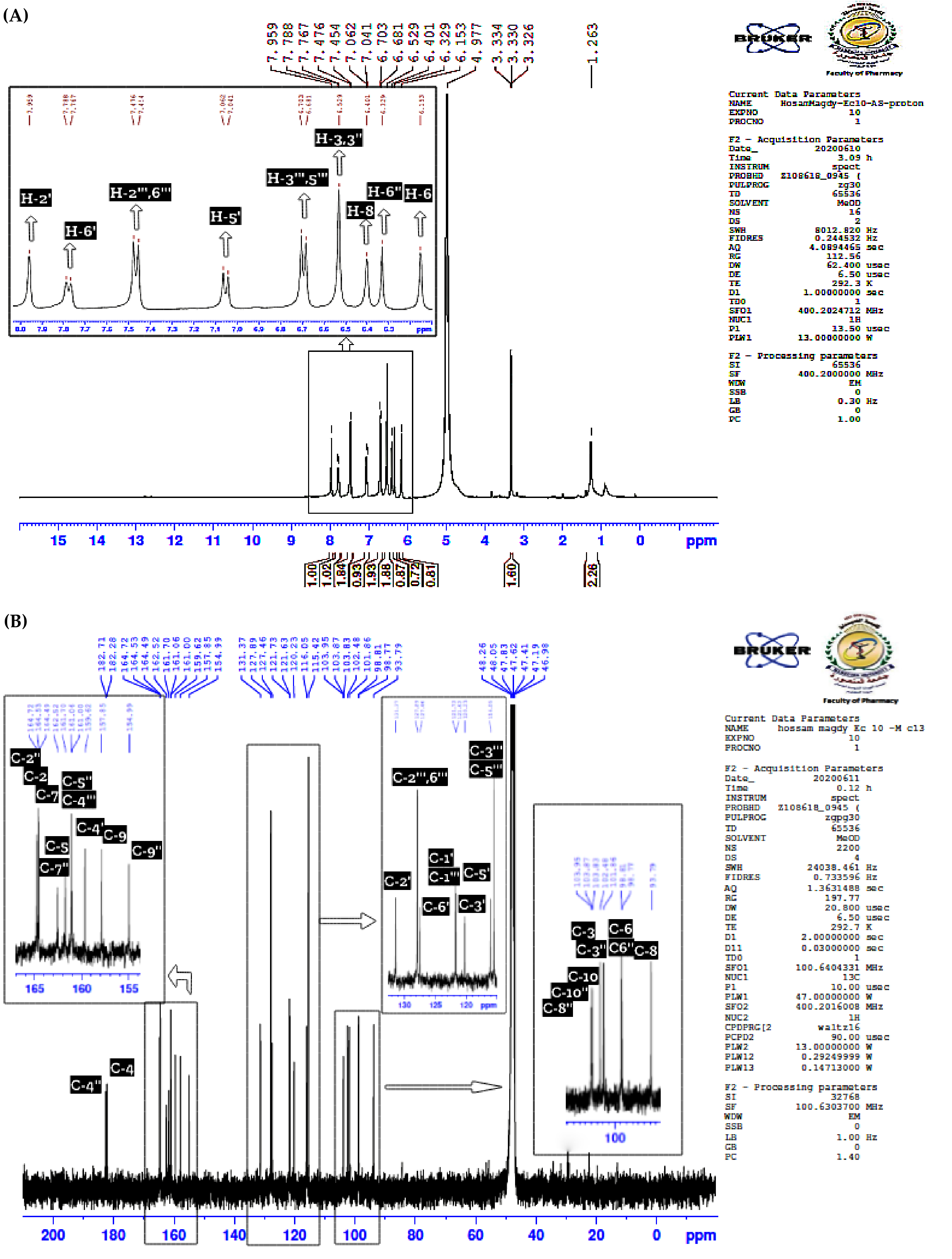

Figure S17. (A) ^{1}H and (B) ^{13}C-NMR spectrum of compound (**5**) (CD_3OD)

(A)

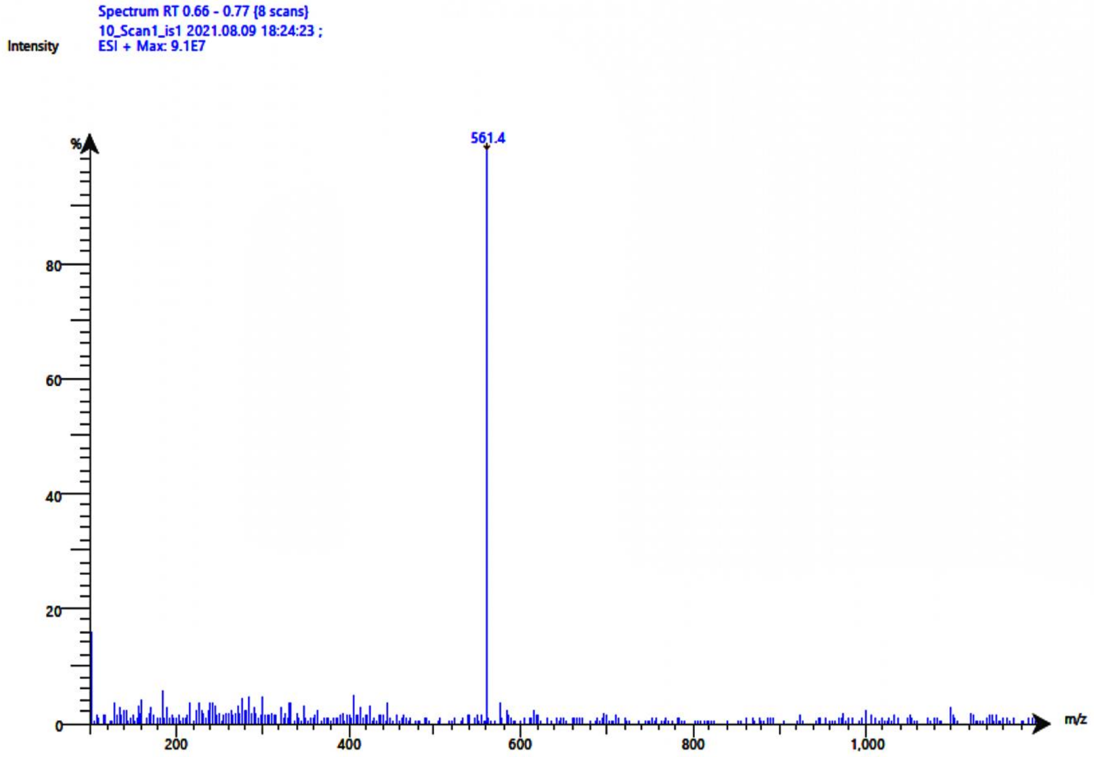

(B)

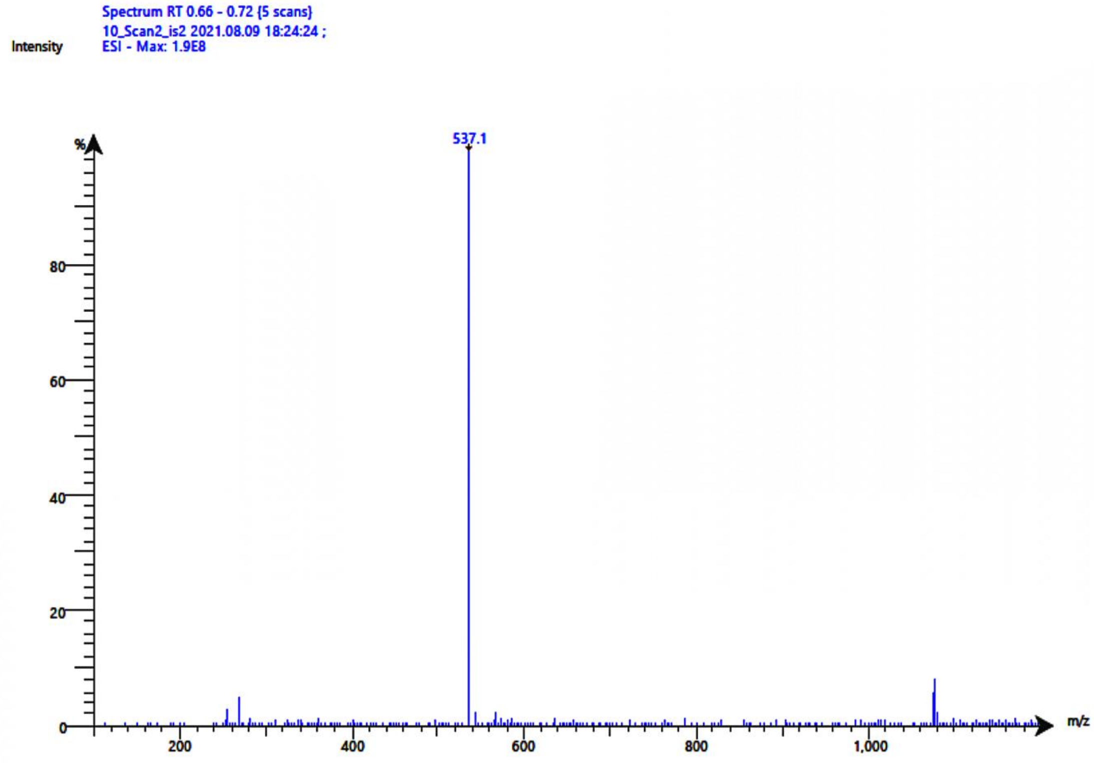

Figure S18. ESIMS ''positive (A) and negative modes (B)'' of compound (**5**)

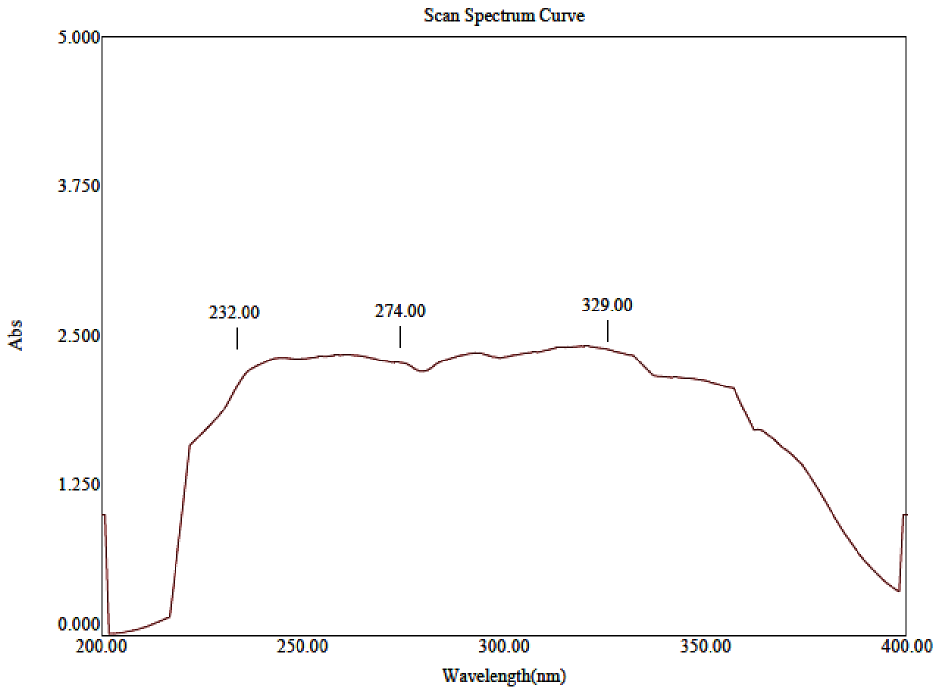

Figure S19. UV spectrum of compound (**5**) in CH_3OH

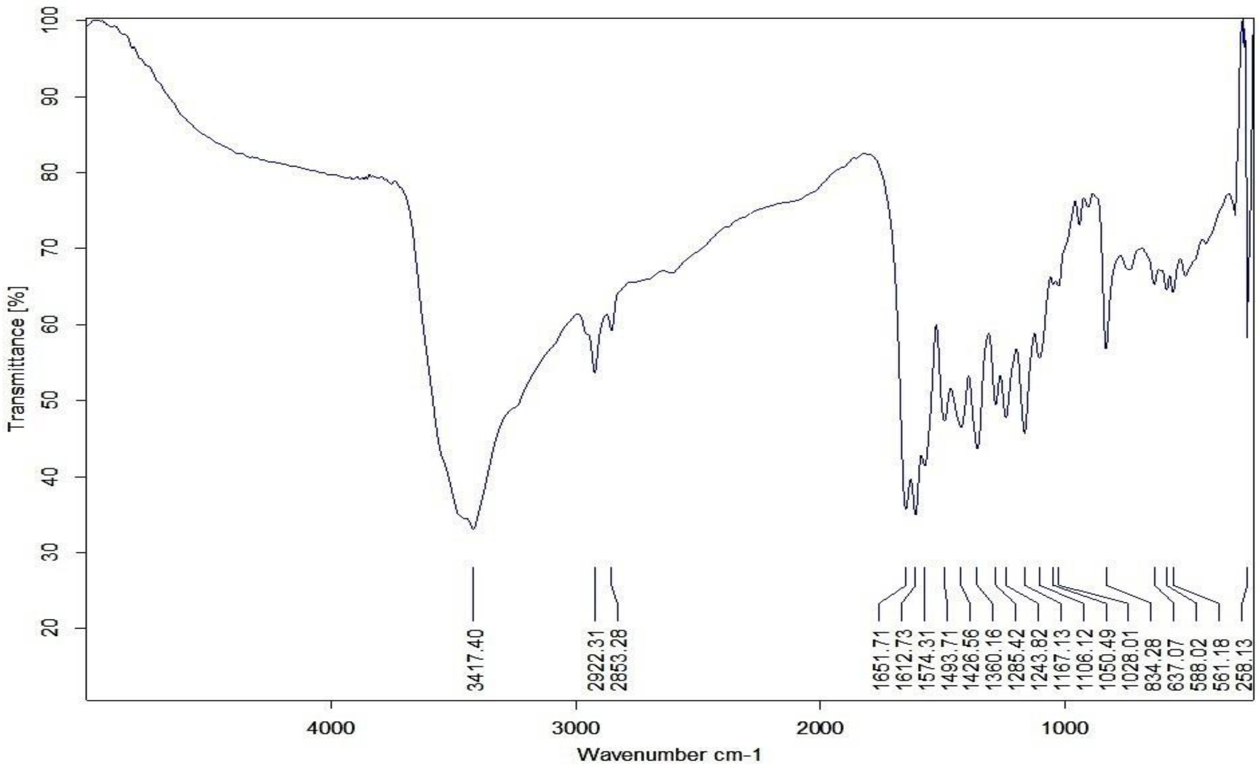

Figure S20. IR spectrum of compound (**5**) in KBr disc

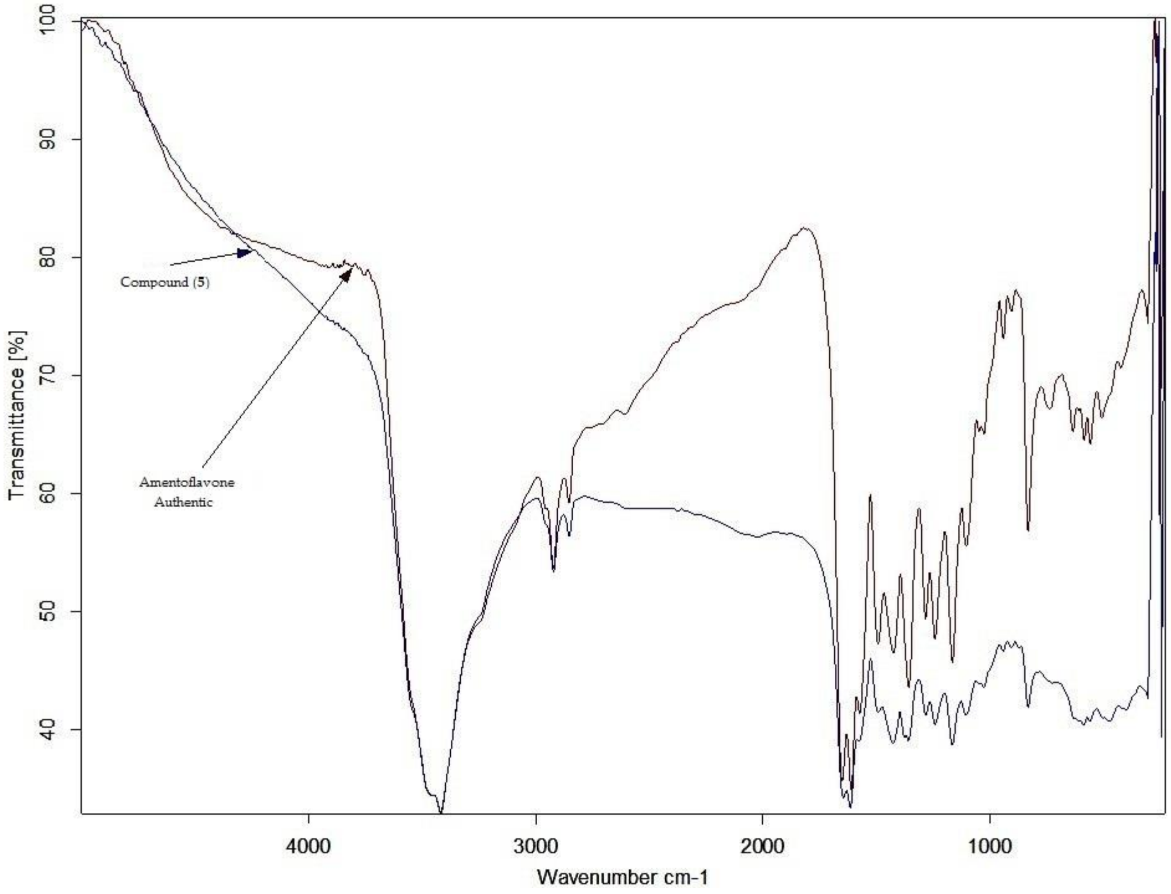

Figure S21. IR fingerprint spectrum of compound (**5**) and amentoflavone authentic sample in KBr disc

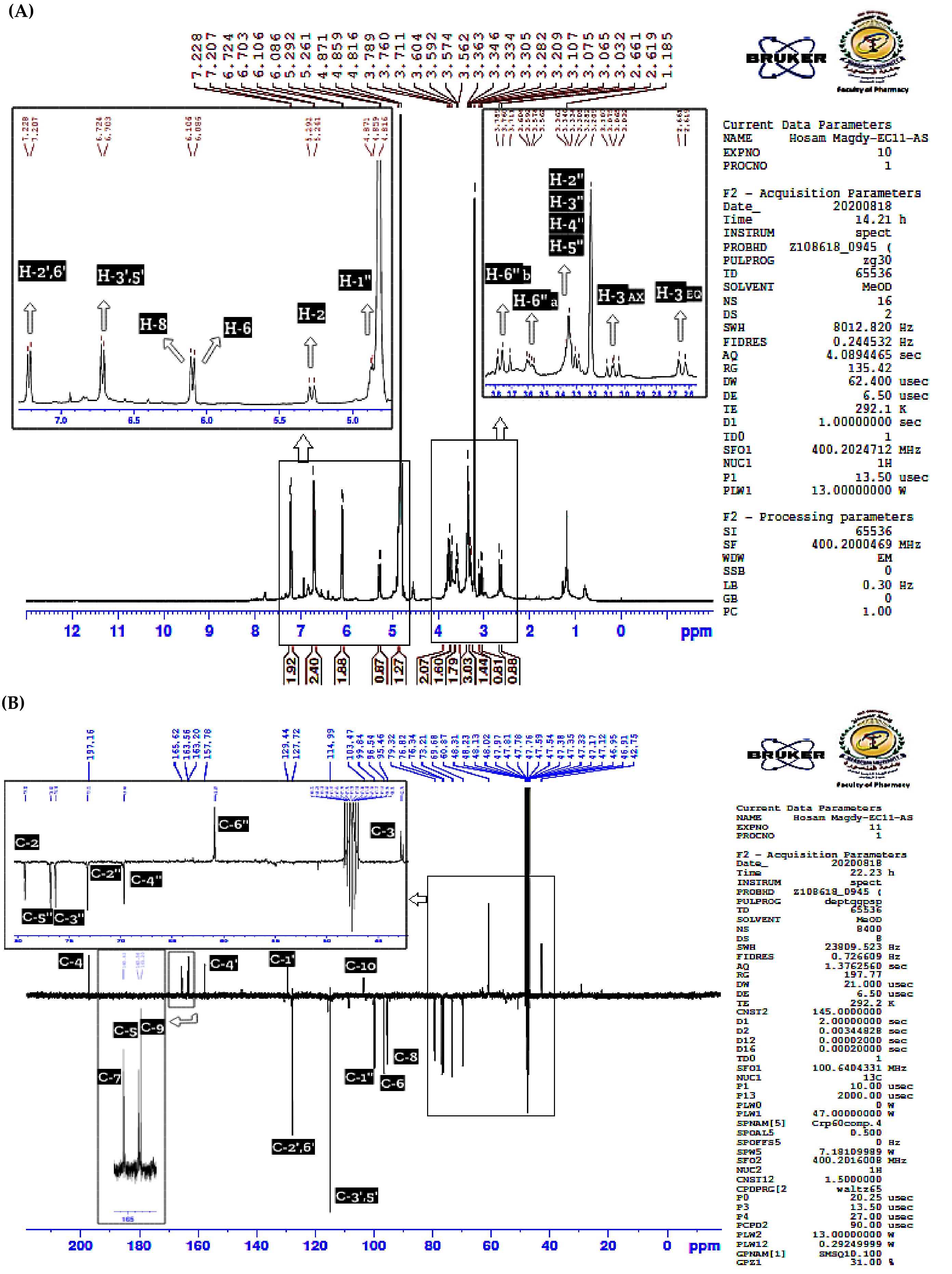

Figure S22. (A) ^{1}H and (B) DEPTQ NMR spectrum of compound (**6**) (CD_3OD)

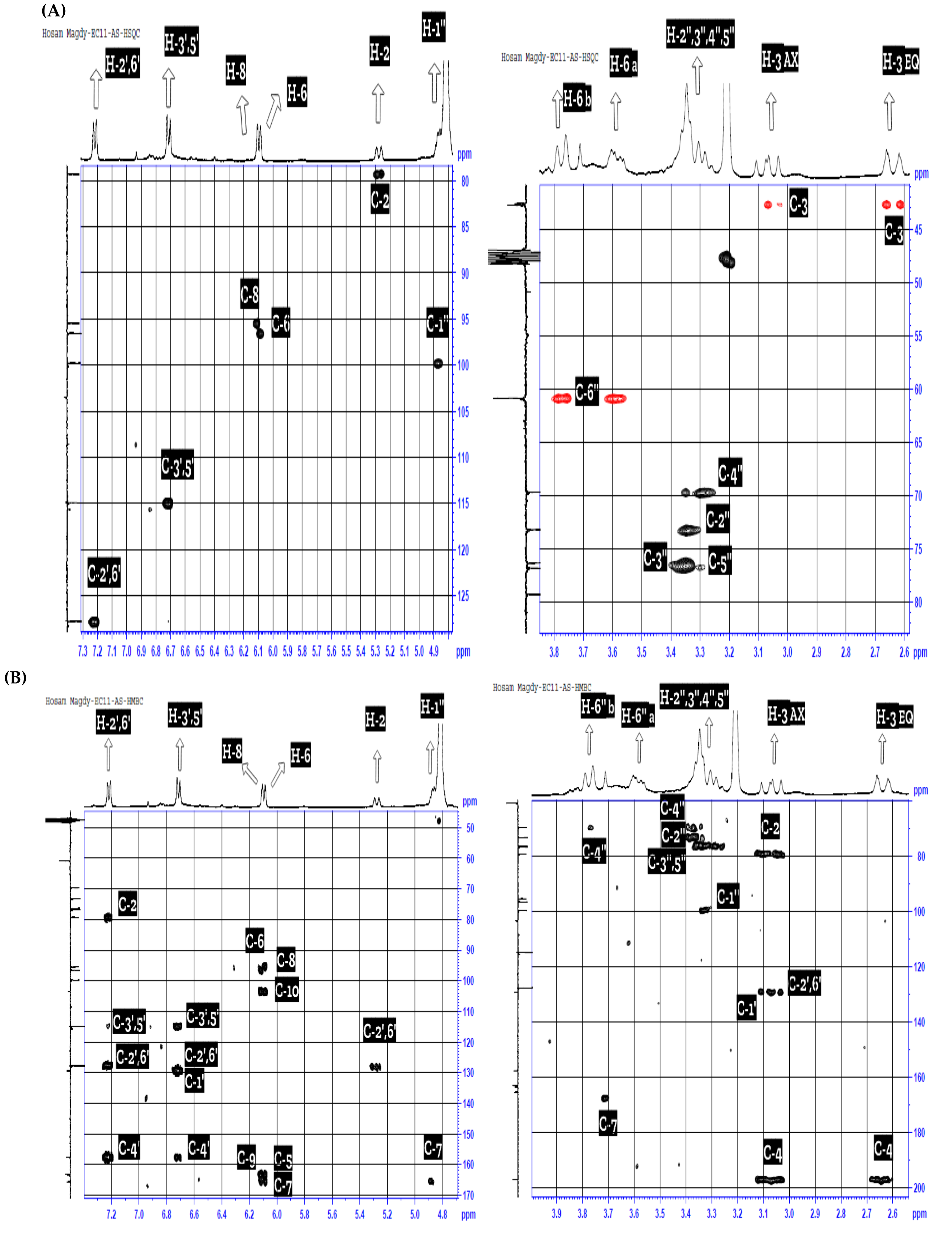

Figure S23. (A) HSQC and (B) HMBC spectrums of compound (**6**) (CD_3OD)

(A)

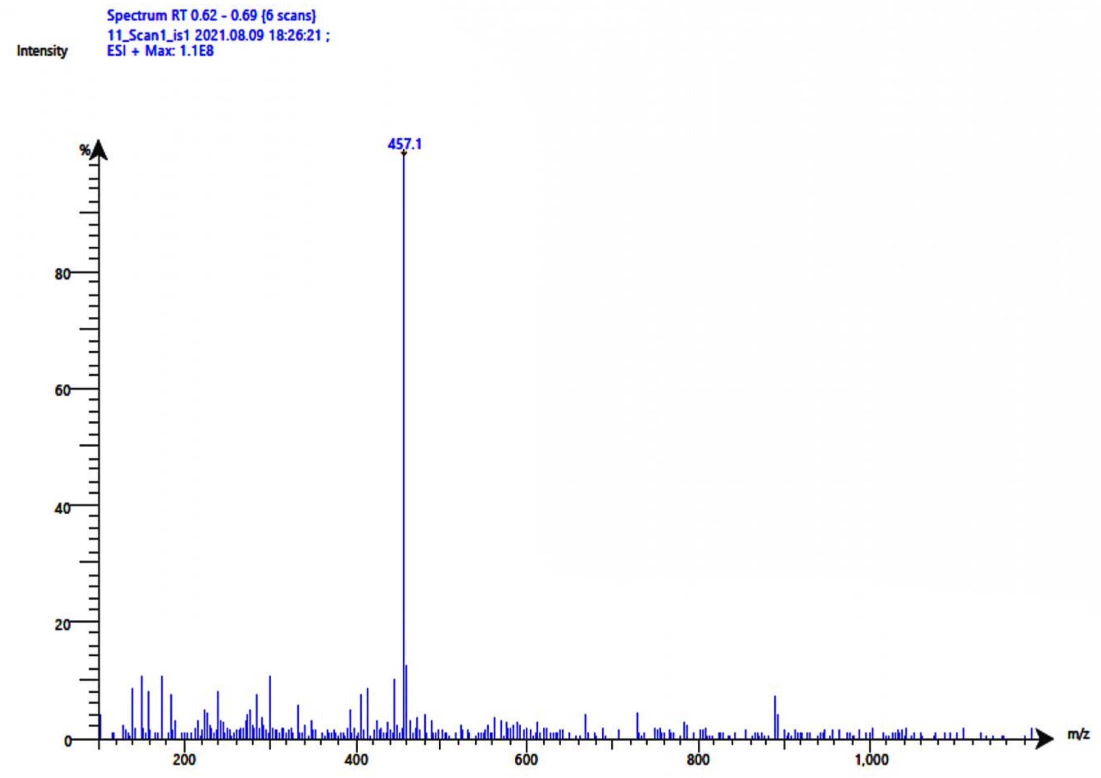

(B)

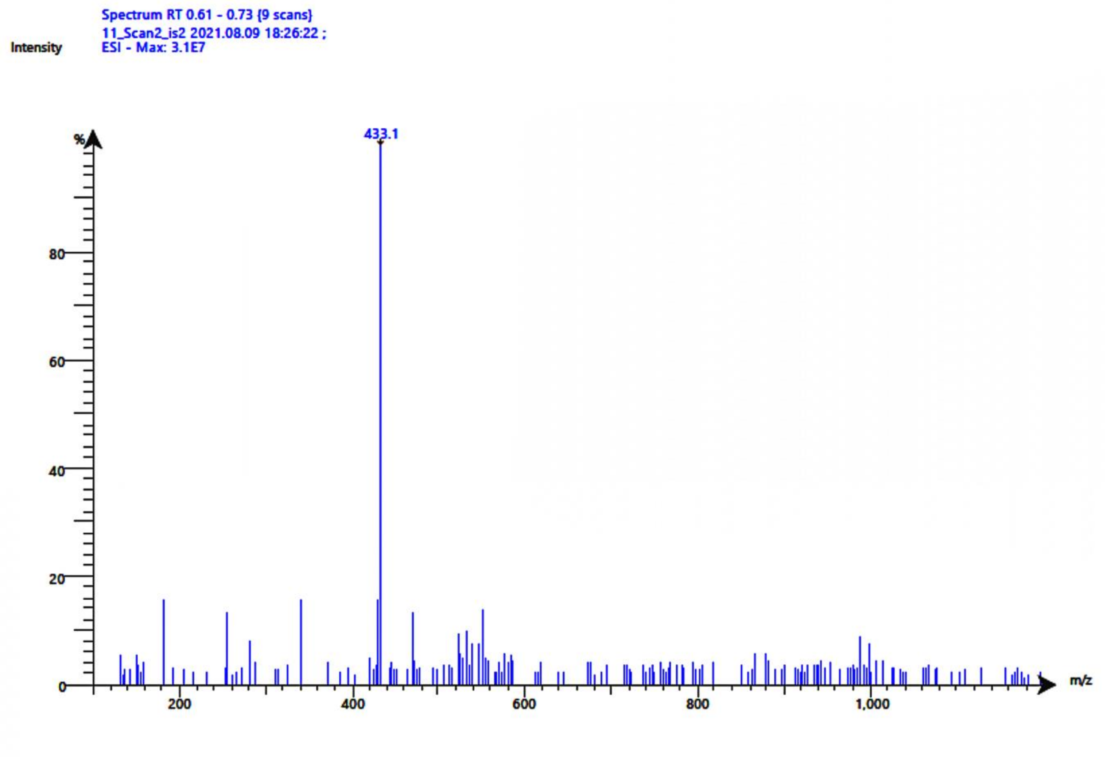

Figure S24. ESIMS "positive (A) and negative modes (B)" of compound (**6**)

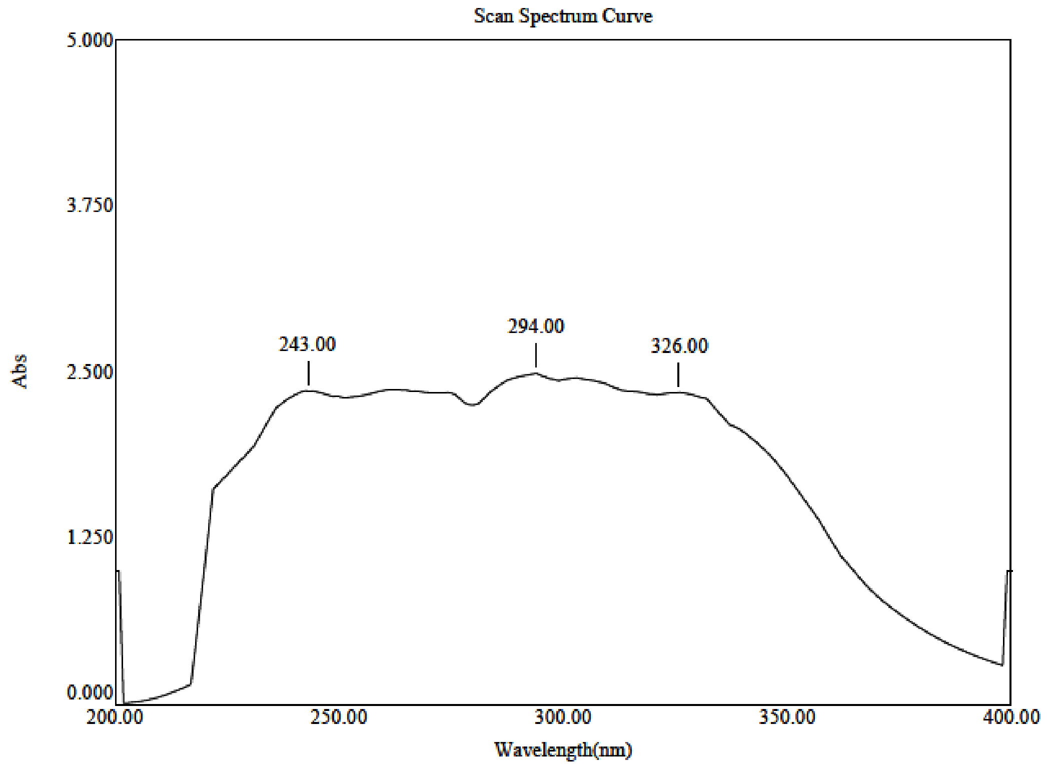

Figure S25. UV spectrum of compound (**6**) in CH_3OH

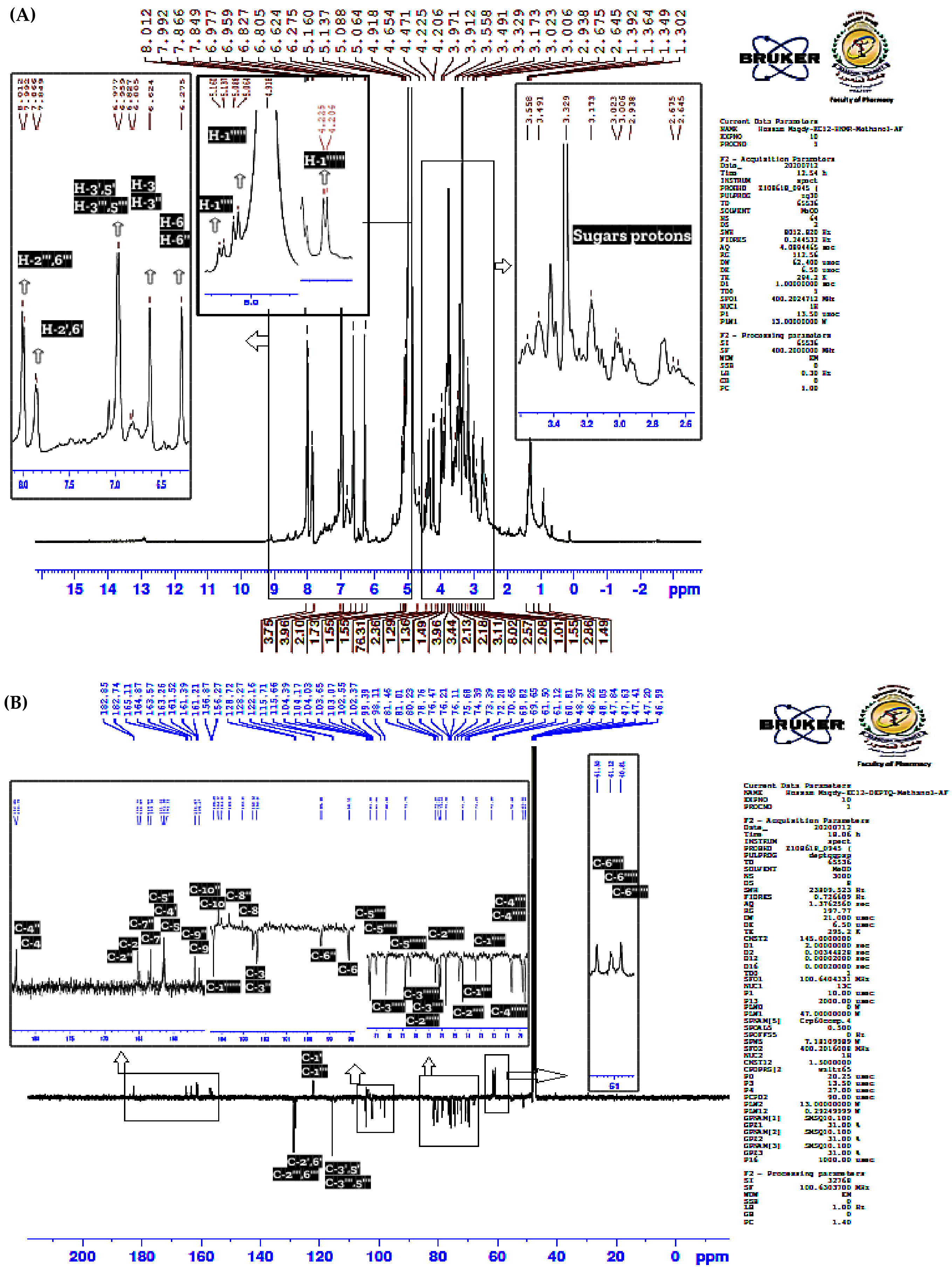

Figure S26. (A) ^{1}H and (B) DEPTQ NMR spectrum of compound (**7**) (CD_3OD)

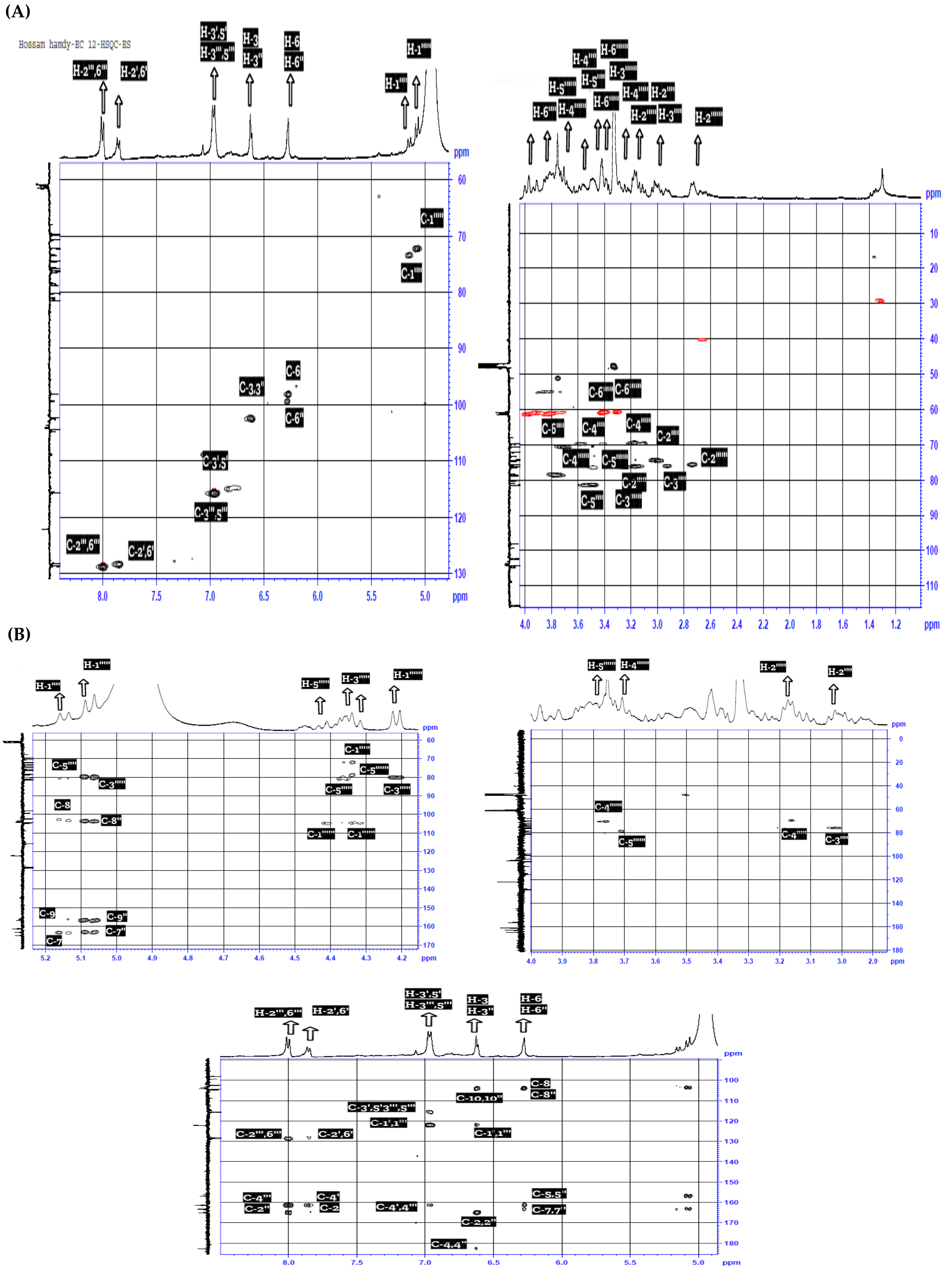

Figure S27. (A) HSQC and (B) HMBC spectrums of compound (**7**) (CD_3OD)

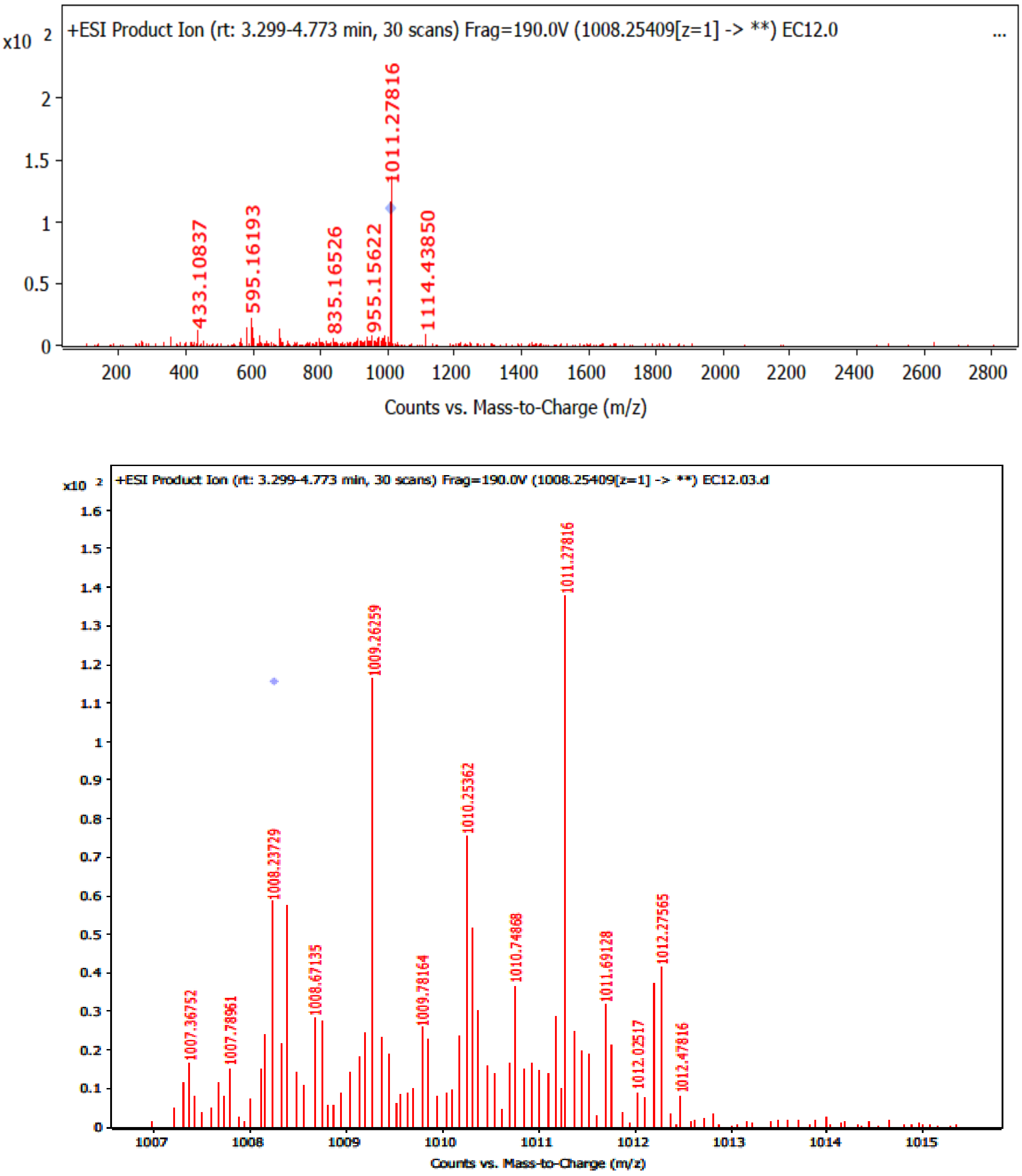

Figure S28. HRMS (ESI+) (positive mode) of compound (**7**) with zooming to molecular ion region

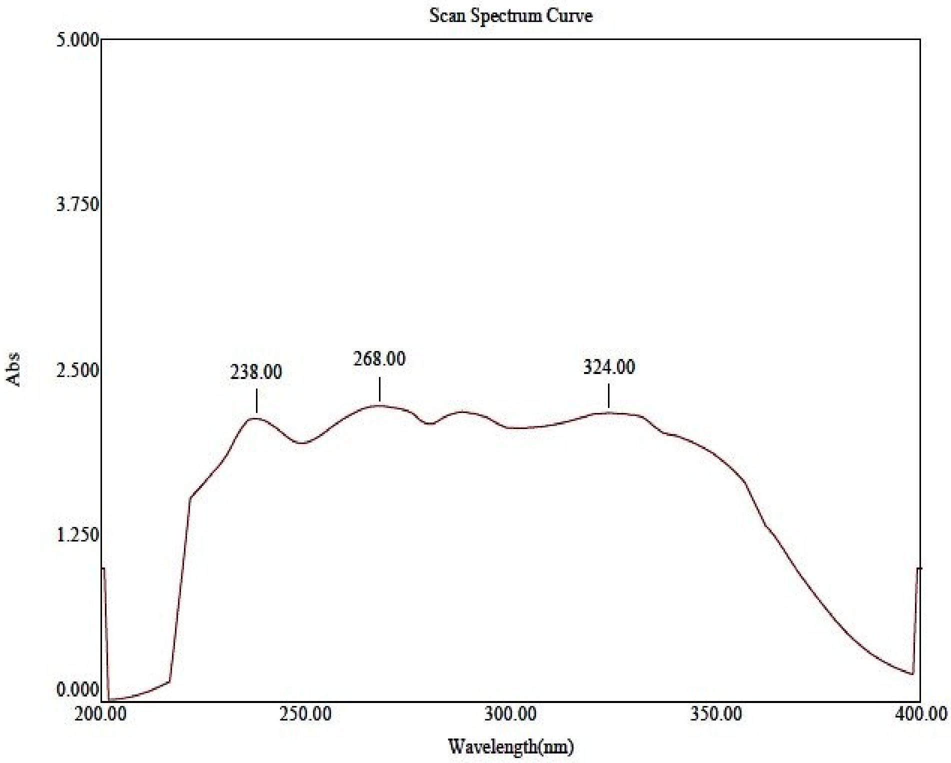

Figure S29. UV spectrum of compound (**7**) in CH_3OH
